# SLC7A11 in Fibrosis: Molecular Mechanisms and Future Prospects

**DOI:** 10.14336/AD.2025.0106

**Published:** 2025-04-05

**Authors:** Juntong Chen, Yanjie He, Ru Chen, Zhiyong Yang, Xiaomeng Luo, Fan Yang

**Affiliations:** ^1^School of Medicine, Zhejiang University, Hangzhou, China.; ^2^Department of Surgery, New York University School of Medicine and NYU-Langone Medical Center, New York, NY 10016, USA.; ^3^Digestive Endoscopy Center, Affiliated Hospital of Nanjing University of Chinese Medicine, Nanjing, China.; ^4^Department of Cardiology, Shengjing Hospital of China Medical University, Shenyang, China.; ^5^Department of Rheumatology and Immunology, The First Hospital of China Medical University, Shenyang, China.; ^6^Department of Gastroenterology, Shengjing Hospital of China Medical University, Shenyang, China.

**Keywords:** SLC7A11, fibrosis, ferroptosis, pathogenic mechanisms

## Abstract

Solute carrier family 7 member 11 (SLC7A11), or xCT, is a cystine-glutamate antiporter crucial for maintaining cellular antioxidant capacity through the uptake of cystine, which is vital for glutathione synthesis. This protein plays an important role in regulating ferroptosis, an iron-dependent form of cell death. Fibrosis, a pathological condition characterized by the excessive accumulation of fibrous connective tissue, is a health challenge because it can lead to organ dysfunction and failure. Emerging evidence links SLC7A11 to the regulation of fibrosis through its involvement in ferroptosis and other mechanisms. This review aims to explore the effects of SLC7A11 on fibrotic diseases in various organs. We will delve into both ferroptosis-dependent and -independent pathways and propose therapeutic strategies that target SLC7A11 to mitigate fibrosis, emphasizing the need for cell-type-specific interventions. This review provides a foundational understanding for the development of targeted treatments involving SLC7A11 for managing fibrotic diseases.

## Introduction

Fibrosis is a pathological process characterized by parenchymal cell necrosis, along with an abnormal extracellular matrix (ECM) production and excessive accumulation in tissues resulting from inflammation. Mild fibrosis may have limited clinical effect, whereas severe fibrosis can cause tissue structure degradation and organ sclerosis. Normal organs consist of two components: parenchyma and stroma. Parenchymal cells (e.g., hepatocytes in the liver) are responsible for organ functions. In contrast, the mesenchyme primarily comprises mesenchymal cells (such as fibroblasts) and ECM (such as collagen), which provide support, protection, connection, and a microenvironment for organ functioning. The mesenchyme also transmits intercellular information in some organs. Complete regeneration becomes impossible when tissues or organs have severe damage (e.g., chronic hepatitis) or non-regenerative cell injury; instead, fiber connective tissue proliferates to repair the damaged tissue, resulting in fibrosis. Fibrosis is a reparative mechanism in response to tissue injury intended to maintain the overall integrity of tissues and organs. Although the increased fibrous connective tissue addresses the damage, it does not have the same structural and functional characteristics as the original parenchymal cells. Consequently, the functioning of most organs declines substantially once fibrosis develops. Thus, fibrosis has both beneficial and harmful effects on organ tissues.

Fibrosis occurs in various organs, such as the liver, heart, lungs, and kidneys, and manifests as a range of diseases. The reduction in the number of parenchymal cells and the increase in the number of stromal cells in fibrotic diseases is primarily responsible for the clinical manifestations of organ dysfunction. Owing to differences in the degree of fibrosis, the clinical manifestations are also different. For example, patients with mild liver fibrosis may show no obvious clinical manifestations; however, as the disease progresses, they may develop clinical manifestations of liver decompensation, such as upper gastrointestinal bleeding, abdominal distention due to ascites, and cognitive or executive function disturbances caused by hepatic encephalopathy. Moreover, treatments to slow the progression of liver fibrosis have limited effect, and the only effective treatment for advanced cirrhosis is liver transplantation; therefore, liver fibrosis has poor prognosis. Additionally, fibrosis of specific tissues or organs may have organ-specific clinical consequences, such as hypertrophic scars affecting appearance or corneal fibrosis, which can lead to blindness.

Recently, the prevalence of fibrotic conditions has increased. Liver disease is responsible for approximately 2 million deaths globally each year, with cirrhosis alone contributing to 1 million of these deaths [1]. In 2010, cirrhosis was responsible for approximately 49,500 deaths in the United States, making it the 8th leading cause of death [2]. Furthermore, liver cancer, which often occurs as a complication of cirrhosis, was estimated to cause 19,500 deaths. Similarly, a study using data from the National Death Index of the US Centers for Disease Control and Prevention (CDC) and the Rochester Epidemiology Project estimated that in 2008, liver disease accounted for 66,007 deaths, with 18,175 of these being attributed to hepatobiliary cancers [3]. Idiopathic pulmonary fibrosis (IPF) is the most prevalent form of pulmonary fibrosis (PF), typically affecting individuals aged 65 years and older. In adults aged over 65 years, the estimated prevalence of IPF is up to 400 cases per 100,000 population [4]. Approximately 3 million individuals globally are affected by IPF, and the incidence in older adults is increasing [5]. The survival rate after diagnosis generally ranges from 3 to 5 years, with a 5-year survival rate of 20-40% [4]. Some studies suggest that the survival rate of patients with IPF is lower than that of many cancers with similar demographic characteristics [6].

### Mechanisms of fibrosis

Fibrotic diseases can be triggered by various factors, such as the reparative process following myocardial infarction, prolonged stimulation from chronic inflammation, and long-term exposure to toxic chemicals. However, regardless of the trigger or the organ affected, the fibrotic process invariably follows a common pathway: activation of fibroblasts, leading to the production of ECM [7].

The excessive production of ECM is influenced by multiple factors, but the activation of fibroblasts is a key event in this process. Among these, transforming growth factor-β (TGF-β) plays a pivotal role. TGF-β plays a crucial role in early embryonic development, organ formation, immune regulation, tissue repair, and maintaining homeostasis in adults [8]. TGF-β must be released from its latent complex to provoke a biological response, referred to as latent TGF-β activation or formation [9]. TGF-β uses the classic signal transducers and transcriptional modulators (SMAD) pathway to exert its biological effects [8]. Activated TGF-β has serine/threonate kinase on the corresponding membrane receptors (TGF-βRⅠ and TGF-βRⅡ), which bind to form receptor complexes [10], ultimately leading to the activation of fibroblasts. The relationship between TGF-β and fibrosis has been reviewed in detail [7, 11, 12].

Various cytokines are potential fibrogenesis triggers. The prevention of fibrosis by inhibiting cytokines, inflammatory factors, and immune cells indicates that immune mechanisms are crucial in the progression of fibrosis [13]. However, the inflammatory response has a bidirectional effect on the occurrence and progression of fibrosis and has the potential to promote or inhibit fibrosis. Moreover, the development of fibrosis does not depend exclusively on the presence of an inflammatory response. A previous review provides further details regarding the association between inflammation and fibrosis [14].

### Relationship between SLC7A11, ferroptosis, and fibrosis

Recently, ferroptosis has emerged as a prominent research area, with studies demonstrating its association with the pathogenesis and progression of fibrotic diseases [15-17]. Fibrosis is a considerable burden on healthcare systems globally, thus making the identification of targets associated with the onset and progression of fibrosis and the development of corresponding therapeutic drugs a primary research focus. Solute carrier family 7 member 11 (SLC7A11, also known as xCT), a ferroptosis regulator, may affect fibrosis through ferroptosis- and non-ferroptosis-dependent pathways. Therefore, targeting SLC7A11 could be a promising therapeutic approach for treating fibrotic diseases. System Xc^-^ is a sodium-independent antiporter, allowing the export of one glutamate molecule to the extracellular space while simultaneously importing one cystine molecule into the cell [18]. System Xc^-^ comprises the light-chain subunit SLC7A11 and the heavy-chain subunit solute carrier family 3 member 2 (SLC3A2, also known as CD98hc or 4F2hc), which is linked by a disulfide bond. SLC7A11 is responsible for the antiporter function of system Xc^-^, whereas SLC3A2 anchors SLC7A11 to the plasma membrane and stabilizes the SLC7A11 protein [19].

Many researchers have investigated the relationship between SLC7A11 and fibrosis. Therefore, this review summarizes these studies to draw preliminary conclusions to guide future research and drug development. It provides a comprehensive overview of the role of SLC7A11 in fibrotic diseases affecting various organs or tissues, including the liver, lung, kidney, heart, pancreas, intestine, keloid, and cornea, describes how SLC7A11 exerts ferroptosis- and non-ferroptosis-dependent effects on fibrosis, and proposes potential therapeutic strategies for fibrotic diseases based on the distinctive characteristics of SLC7A11.

**Figure 1.**
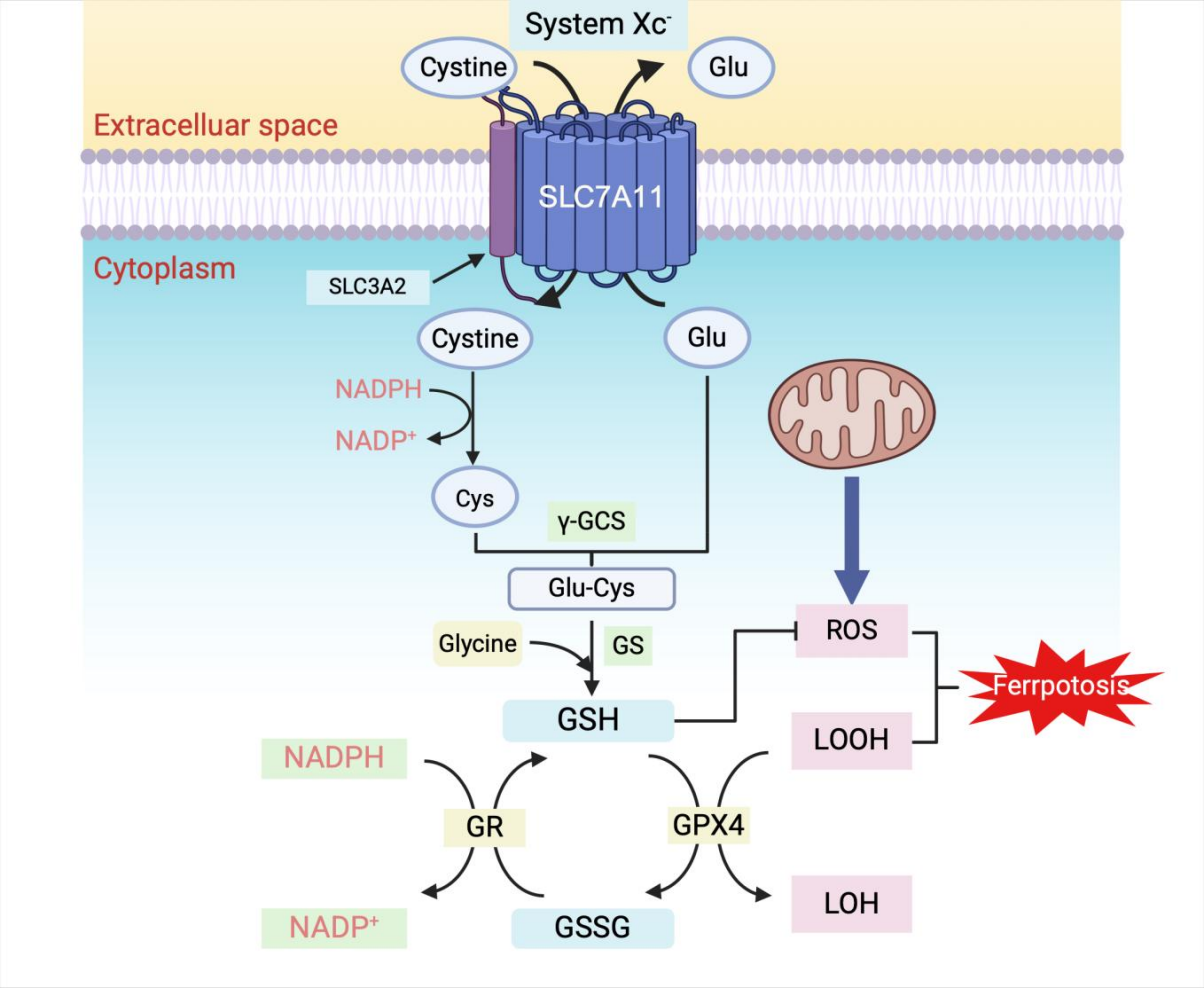
**Function of solute carrier family 7 member 11**. System Xc^-^ is a cystine/glutamate transporter that imports one cystine molecule into the cell in exchange for the export of one glutamate molecule. This system comprises two subunits: the light-chain subunit solute carrier family 7 member 11 (SLC7A11), which is responsible for the primary function of System Xc^-^, namely, the transport of cystine and glutamate, and the heavy-chain subunit solute carrier family 3 member 2 (SLC3A2), which anchors SLC7A11 to the cell membrane and maintains its stability. Once extracellular cystine is transported into the cell, it is reduced by NADPH to cysteine. Cysteine, catalyzed by γ-glutamylcysteine synthetase (γ-GCS), then combines with glutamate to form γ-glutamylcysteine. This intermediate is combined with glycine by glutathione synthetase (GS) to produce biologically active glutathione (GSH). Ferroptosis is a type of iron-dependent programmed cell death that is regulated by amino acid availability, with GSH playing a crucial role as an antioxidant. GSH prevents ferroptosis by reducing lipid peroxides (LOOH) or by reducing the accumulation of reactive oxygen species (ROS). Glutathione peroxidase 4 (GPX4) uses GSH to reduce LOOH or ROS into their inactive forms (LOH or ROH), thereby preventing ferroptosis. After GSH is used, it is oxidized to glutathione disulfide (GSSG), which can be reduced back to GSH by NADPH, allowing it to continue to prevent ferroptosis.

### Function of SLC7A11

SLC7A11 plays a key role in cystine metabolism. Cystine, the primary extracellular form of cysteine, is transported into cells by SLC7A11, where it is reduced to cysteine. The intracellular reducing agent nicotinamide adenine dinucleotide phosphate (NADPH) plays an important role in maintaining the intracellular reducing environment and supports cystine uptake mediated by SLC7A11. Cysteine is essential for maintaining cellular homeostasis and plays multiple roles within the cell, including catalysis, transport, and mediating oxidative stress responses [20]. Intracellular cysteine can be recycled by de novo biosynthesis via the trans-sulfur pathway or protein degradation. However, de novo cysteine synthesis is insufficient for cell survival under certain conditions, particularly under conditions of oxidative stress [21]. Therefore, SLC7A11-dependent cystine transport is crucial for cells especially under stress conditions.

An intracellular cysteine deficiency disrupts cellular functions, including those involved in ferroptosis. Ferroptosis is a recently identified type of iron-dependent programmed cell death that differs from apoptosis, necrosis, and autophagy [22]. The primary mechanism of ferroptosis is lipid peroxidation catalysis of highly expressed unsaturated fatty acids on the cell membrane under the influence of bivalent iron or ester oxygenase, thereby inducing cell death [23]. Intracellular redox homeostasis ensures a balance between oxidation and reduction reactions within the cell. When this balance is disrupted, oxidative stress occurs, leading to the excessive production of reactive oxygen species (ROS) and lipid hydroperoxides (LOOH), which can ultimately trigger ferroptosis. Ferroptosis is strongly influenced by amino acid metabolism, particularly by glutathione (GSH), a tripeptide composed of cysteine, glutamate, and glycine. GSH is the primary intracellular antioxidant [19] and plays a crucial role in reducing ROS and LOOH to prevent ferroptosis. However, the synthesis of GSH is limited by the availability of cysteine, and SLC7A11 regulates cysteine content to ensure adequate GSH production, thereby preventing ferroptosis. In addition, GSH peroxidase (GPX4) plays a protective role during ferroptosis by acting as an important cofactor for ROS reduction by GSH [23, 24]. The intracellular function of SLC7A11 is summarized in Figure 1. In addition to the influence of amino acids, ferroptosis is intricately linked to iron and lipid metabolism [25-27]. Several drugs can affect the process of ferroptosis. Erastin inhibits SLC7A11-mediated cystine import, thereby inducing ferroptosis [22, 28]. In contrast, ferrostatin-1 (Fer-1) is a ferroptosis inhibitor that prevents ferroptosis by inhibiting lipid peroxidation and is widely used in various ferroptosis-related studies [29].

### Regulatory mechanisms of SLC7A11

**Figure 2.**
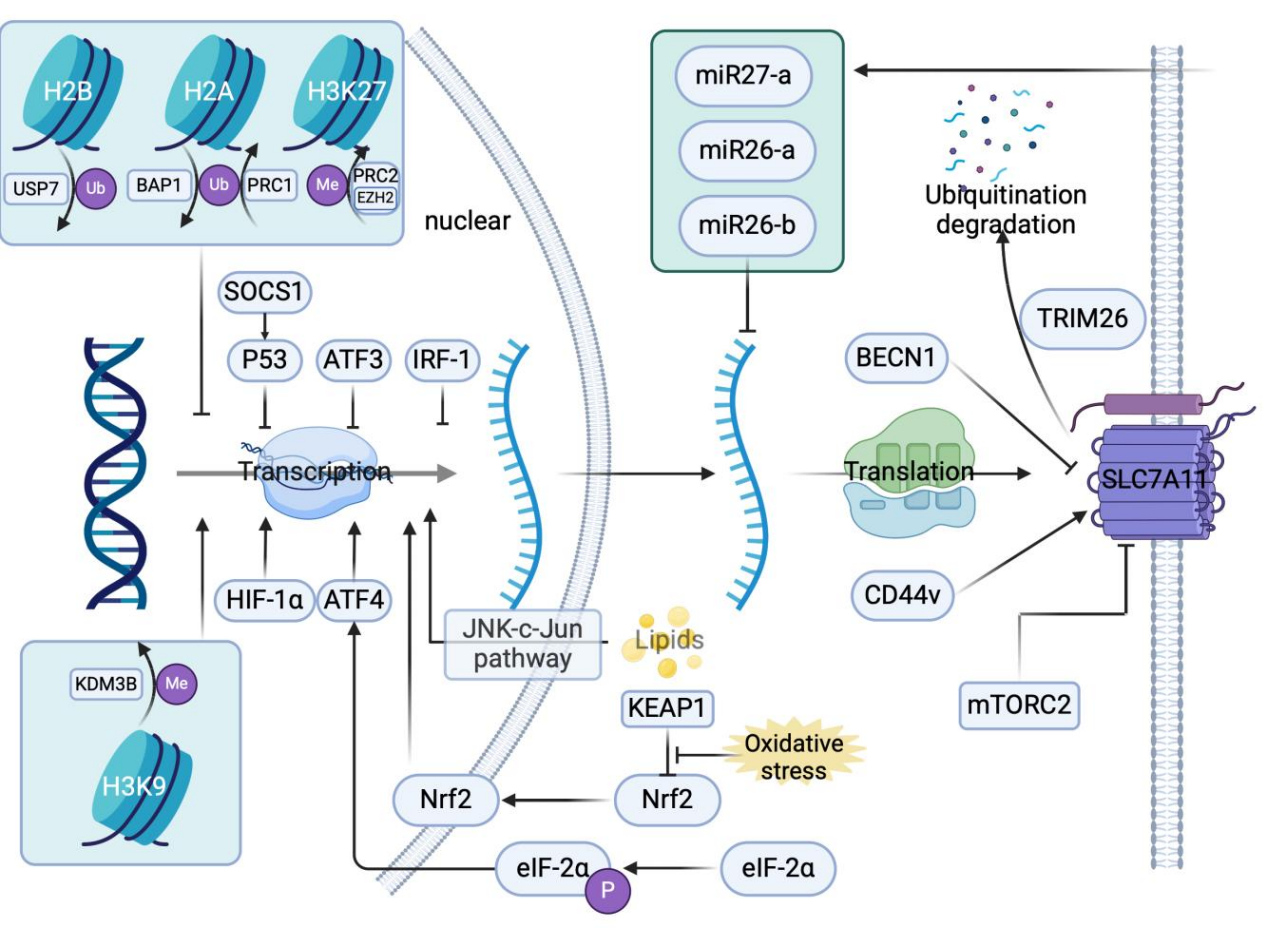
**Regulatory mechanisms of SLC7A11**. At the epigenetic level, BRCA1-associated protein 1 (BAP1) promotes the deubiquitination of histone H2A, whereas polycomb repressive complex 1 (PRC1) facilitates H2A ubiquitination, both of which can suppress SLC7A11 expression. Enhancer of zeste homolog 2 (EZH2), as part of polycomb repressive complex 2 (PRC2), induces histone H3 at lysine 27 (H3K27) methylation and ubiquitin-specific-processing protease 7 (USP7) promotes the deubiquitination of histone H2B, both of which can reduce SLC7A11 expression. Conversely, lysine demethylase 3B (KDM3B) demethylates H3K9, thereby promoting SLC7A11 expression. At the transcriptional level, suppressor of cytokine signaling 1 (SOCS1) inhibits SLC7A11 transcription via tumor suppressor protein p53 (p53) and activating transcription factor 3 (ATF3) and interferon regulatory factor 1 (IRF-1) also suppress SLC7A11 transcription. Under oxidative stress, inhibition of nuclear factor erythroid 2-related factor 2 (Nrf2) by Kelch-like ECH-associated protein 1 (KEAP1) is prevented, leading to SLC7A11 transcription activation. Phosphorylated eukaryotic initiation factor 2α (eIF-2α) activates SLC7A11 transcription through activating transcription factor 4 (ATF4), and hypoxia-inducible factor 1 alpha (HIF-α) also activates SLC7A11 transcription. Lipids activate SLC7A11 transcription via the c-Jun N-terminal kinase (JNK-c-Jun) pathway. At the mRNA level, exosome-derived miR-27a, miR-26a, and miR-26b inhibit SLC7A11 mRNA activity. At the post-translational level, SLC7A11 can be ubiquitinated and degraded by tripartite motif-containing 26 (TRIM26). Beclin 1 (BECN1) inhibits SLC7A11 activity by binding to it, whereas the mechanistic target of rapamycin complex 2 (mTORC2) suppresses SLC7A11 activity. Variant isoforms of CD44 (CD44v) stabilizes SLC7A11.

SLC7A11 is regulated by a variety of mechanisms, including epigenetic, transcriptional and post-translational regulation (Fig. 2). We describe the regulation of *SLC7A11* gene expression in the sequential order of its progression from gene to protein formation.

### Epigenetic regulation of SLC7A11 (DNA and histone modifications)

Epigenetic regulation is the process of regulating the content and function of intracellular nucleic acids or proteins through epigenetic modifications (such as methylation, acetylation, and phosphorylation) [30]. The first aspect of epigenetic regulation involves DNA and histone modifications, which influence the transcription of SLC7A11, thereby regulating its gene expression. BRCA1 associated protein 1 (BAP1) is a nuclear deubiquitinating enzyme that removes mono-ubiquitylation (H2Aub) from histone 2A at lysine 119, a histone modification that normally inhibits transcription [31]. Polycomb repressive complex 1 (PRC1) is a major ubiquitin ligase that regulates H2Aub [32]. However, both BAP1 and PRC1 inhibit SLC7A11 expression [32, 33], possibly through the dynamic balance of H2A ubiquitination and deubiquitination under BAP1 and PRC1 regulation [19]. Although H2Aub is related to transcriptional repression, mono-ubiquitination of histone 2B (H2Bub) at lysine 120 is typically associated with transcriptional activation. p53 negatively regulates H2Bub1 levels independently of p53 transcription factor activity by promoting the nuclear translocation of the deubiquitinase, ubiquitin-specific-processing protease 7, resulting in SLC7A11 transcriptional repression [34]. Enhancer of zeste homolog 2 (EZH2), a component of polycomb repressive complex 2 (PRC2), facilitates histone H3 trimethylation at lysine 27 (H3K27me3), thereby repressing downstream genes, including SLC7A11 [35]. H3K9 methylation is associated with transcriptional repression [36]. The overexpression of lysine demethylase 3B (KDM3B), an H3K9 demethylase, inhibits H3K9 methylation and promotes SLC7A11 expression [37].

Epigenetic modifications, such as histone methylation, regulate SLC7A11 expression. Proteins such as BAP1 and PRC2 influence this regulation, which in turn affects ferroptosis, which is critical to the pathogenesis of fibrosis. Yan et al. [35] showed that EZH2 negatively regulates SLC7A11 expression in an epigenetic manner, which in turn promotes the progression of renal fibrosis. These modifications provide a mechanism for changes in cellular function that promotes fibrotic progression.

### Transcriptional regulation of SLC7A11

Various transcription factors participate in the transcriptional regulation of SLC7A11, modulating SLC7A11 expression. Activating transcription factor 4 (ATF4) is a transcription factor involved in the integrated stress response (ISR). Nuclear factor erythroid 2-related factor 2 (NRF2) is a key regulator of cellular antioxidant responses [38]. ATF4 and NRF2 play key roles in transcriptional regulation and usually function synergistically as regulators of stress-induced genes [19]. NRF2 is normally expressed in small amounts in various cells and has been linked to the control of NRF2 ubiquitination and proteasomal degradation by three E3 ubiquitin ligase complexes [39]. Under amino acid starvation, kinases such as GCN2 phosphorylates eukaryotic initiation factor-2α, which attenuates Cap-dependent translation while promoting the preferential translation of ISR-specific mRNAs, such as ATF4 [40]. ATF4 promotes the transcription of amino acid metabolism-related genes through downstream amino acid reaction elements, including SLC7A11 [41]. The master E3 ubiquitin ligase, KEAP1-CUL3-RBX1, can be damaged by oxidative stress and loses its ability to regulate NRF2, thereby promoting NRF2 transcription of genes that play a role in antioxidant defense and redox maintenance, including SLC7A11 [38]. SLC7A11 is a negative transcriptional target of tumor suppressor protein p53 (p53) [42]; however, SLC7A11 transcription can also be suppressed when the transcription factor ATF3 combines with the SLC7A11 promoter. ATF3 is upregulated by erastin, thereby suppressing SLC7A11 expression [43]. NRF2 and p53 can regulate SLC7A11 expression, thereby affecting the progression of fibrotic diseases. In addition, lipids upregulate SLC7A11 expression by activating the SLC7A11 promoter via the JNK-c-Jun pathway [44]. The transcription factor hypoxia-inducible factor 1-alpha (HIF-1α) affects the progression of fibrotic diseases by upregulating SLC7A11 [45, 46].

Transcriptional regulation of SLC7A11 plays a crucial role in the response to oxidative stress and ferroptosis. Both NRF2 and ATF4 are involved in regulating SLC7A11. Zhang et al. [47] proposed that the downregulation of SLC7A11 expression in cardio-myocytes by p53 may lead to the development of fibrosis. Because SLC7A11 regulates cysteine and glutathione levels, its transcriptional regulation influences cellular redox balance, which can affect fibrotic disease progression through ferroptosis.

### Regulation of SLC7A11 at the mRNA level

Post-transcriptionally, SLC7A11 mRNA is regulated by various factors, including multiple small interfering RNAs (siRNAs) and microRNAs (miRNAs). Notably, this regulatory process is also a part of epigenetic regulation. For regulation at the mRNA level, the exosomal miR-26a from bone marrow mesenchymal stem cells directly binds to SLC7A11 mRNA and reduces the SLC7A11 level in LX-2 human hepatic stellate cells (HSCs) [48]. SLC7A11 mRNA is additionally regulated by non-sense-mediated mRNA decay and miRNAs [49].

### Post-translational regulation of SLC7A11

SLC7A11 is involved in many regulatory pathways after translation. Tripartite motif-containing protein 26 (TRIM26) promotes SLC7A11 degradation by ubiquitination [50]. Mammalian target of rapamycin complex 2 (mTORC2) comprises several protein components, including mTOR, and is a serine/threonine kinase. It primarily influences cell proliferation and survival through the phosphorylation of various members of the AGC (PKA/PKG/PKC) protein kinase family [51]. mTORC2 is an SLC7A11-binding protein [49] and phosphorylates serine 26 in the cytoplasmic N-terminal tail of SLC7A11 in response to growth factors, inhibiting SLC7A11 activity [52]. Variant isoform of CD44 (CD44v) is an adhesion molecule expressed in cancer stem-like cells that interact with xCT and maintain SLC7A11 stability [53]. Beclin 1 (BECN1), also known as ATG6, has various functions that are independent of autophagy. BECN1-SLC7A11 complex formation results in reduced SLC7A11 levels [54]. Additional post-translational regulatory pathways are discussed in the review by Koppula et al. [19], post-translational modifications, such as phosphorylation and ubiquitination, regulate SLC7A11 stability and activity. The TRIM26 protein is particularly important in regulating SLC7A11 activity post-translationally, whereas BECN1-SLC7A11 complex formation downregulates SLC7A11 activity. These mechanisms affect the progression of fibrosis [50, 54].

SLC7A11 has numerous regulatory pathways; however, only a few pathways that may be related to fibrotic diseases are discussed in this review [19, 26]. Some cytokines may be related to the regulation of SLC7A11. Table 1 summarizes the relationship between cytokines, immune cells, SLC7A11, and fibrosis.

### Effects of SLC7A11 on liver fibrosis

As in most organs, fibrosis in the liver is morphologically defined by excessive ECM deposition. Specifically, the structure of liver lobules becomes disordered, and fibrous septa surround regenerating hepatocytes, resulting in pseudolobule formation. Liver fibrosis is marked by nodules of regenerating hepatocytes and the absence or deviation of central veins in the pseudolobules [55].

HSCs are key contributors to the progression of liver fibrosis [56]. The interaction between TGF-β and HSCs plays a crucial role in liver fibrosis. TGF-β promotes the synthesis and suppresses the degradation of ECM proteins, including type I and type II collagen. TGF-β also activates HSCs, the most potent fibrosis agonist [57]. As mentioned previously, activated TGF-β binds to TGF-βRII, leading to the phosphorylation and conformational change of TGF-βRI. Subsequently, TGF-βRI phosphorylates SMAD2 and SMAD3 proteins. Phosphorylated SMAD2 and SMAD3 then form a complex with SMAD4 and translocate to the nucleus, where they regulate the transcription of various genes [58]. This process plays a crucial role in the activation of HSCs. For example, activated SMAD3 can enhance the production of type I and III collagen [59]. Additionally, TGF-β can also activate HSCs via non-SMAD pathways, such as the mitogen-activated protein kinase (MAPK) signaling pathways [58]. Under the stimulation of profibrotic factors such as TGF-β, HSCs are activated into proliferative myofibroblastic hepatic stellate cells (MF-HSCs). MF-HSCs express α-smooth muscle actin, migrate to tissue repair sites, and secrete substantial amounts of ECM [60]. Therefore, reducing MF-HSC activity or preventing HSC transformation into MF-HSCs is an essential target for treating liver fibrosis.

**Table 1:** Cytokines, SLC7A11 and fibrosis.

Cytokines and Immune cells	Relationship with SLC7A11	Model	Association with fibrosis	Ref
TGF-β	SLC7A11 inhibits TGF-β induced HLF overproliferation	HLFs	Positive correlation	[120]
	**SLC7A11 is downregulated during TGF-induced EMT in A549 cells**	A549	Positive correlation	[129]
IL-1β	In the MI model, IL-1β is upregulated, accompanied by the downregulation of SLC7A11	MI (rats)	Positive correlation	[198]
	**SLC7A11 inhibits IL-1 production by reducing the inflammasome NLRP3 expression**	NASH (rats)	Positive correlation	[44]
	**In the angiotensin II-induced atrial fibrosis model, IL-1 is upregulated, accompanied by the downregulation of SLC7A11**	AngII (rats)	Positive correlation	[199]
IL-6	The same as that of IL-1β	MI (rats)	Positive correlation	[198]
	**The same as that of IL-1β**	AngII (rats)	Positive correlation	[199]
TNF-α	In patients with advanced liver cirrhosis, SLC7A11 is downregulated in CD14+ monocytes, accompanied by increased TNF expression, leading to immune dysfunction	Clinical research	Positive correlation	[97]
	**The same as that of IL-1β**	AngII (rats)	Positive correlation	[199]
Macrophages	Downregulation of SLC7A11 in macrophages promotes ferroptosis	BLM (rats)	Positive correlation	[120]
	**Downregulation of SLC7A11 in macrophages promotes ferroptosis**	CCl_4_ (rats)	Negative correlation	[92]

BLM, bleomycin; CCl_4_, carbon tetrachloride; EMT, epithelial-mesenchymal transition; HLF, human lung fibroblast; IL, interleukin; MI, myocardial infarction; NASH, nonalcoholic steatohepatitis; NLRP3, NOD-like receptor protein 3; SLC7A11, solute carrier family 7 member 11; TGF-β, transforming growth factor-β; TNF-α, tumor necrosis factor-α

The inflammatory response and associated immune cells in the liver play a crucial role in the progression of hepatic fibrotic diseases. Kupffer cells are primary mediators and recruit macrophages, neutrophils, dendritic cells, and T lymphocytes, which contribute to fibrosis. [56] Macrophage-secreted TGF-β activates HSCs and stimulates collagen synthesis. [57] Additionally, hepatic macrophages, including Kupffer cells and recruited macrophages, support HSC survival to augment liver fibrosis [61]. Several other cytokines also play crucial roles in liver fibrosis. Interleukin 1β (IL-1β), produced by macrophages, enhances hepatic inflammation by recruiting myeloid cells and activating HSCs, thereby promoting increased collagen production and facilitating liver fibrosis development [62]. IL-13, IL-17, IL-22, and IL-23 affect intrahepatic fibrosis by modulating HSC activity [63-66]. Thus, the inflammatory response is essential for the advancement of liver fibrosis.

In addition, platelet-derived growth factor (PDGF), which is produced by platelets, macrophages, myofibroblasts, and hematopoietic stem cells, exerts a potent mitogenic effect on hematopoietic stem cells. PDGF expression increases fibrotic livers, where it acts synergistically with TGF-β to promote fibrogenesis [67]. Furthermore, hepatocytes are integral to liver fibrosis progression. In summary, the interplay among various cell types such as HSCs, hepatocytes, and inflammatory cells (e.g., TGF-β-producing macrophages to activate HSCs), within liver tissue contributes to liver fibrosis. The reviews by Higashi et al. [68], Parola and Pinzani [69], and Kisseleva and Brenner [70] provide further information on liver fibrosis.

The mechanism of liver fibrosis is intricate and complex, with multiple cell types working together to promote the progression of fibrosis. We examine the effects of SLC7A11 on liver cells or HSCs, and the interactions between different cell types mediated by SLC7A11, focusing on its role in liver fibrosis. Furthermore, we explore the potential for developing SLC7A11-targeted therapeutics for liver fibrosis, and the future directions for research on the effect of SLC7A11 on liver fibrosis.

### SLC7A11 downregulation in HSCs promotes HSC ferroptosis and improves fibrosis

#### Targeting SLC7A11 may promote HSC ferroptosis and improve liver fibrosis

Inducing ferroptosis in HSCs is a promising therapeutic strategy for preventing liver fibrosis. SLC7A11 activity is crucial for MF-HSC function [71], and the promotion of HSC ferroptosis reduces liver fibrosis [72-75]. Recent studies have shown that inducing ferroptosis in HSCs can improve fibrosis [15, 76]. Magnesium isoglycyrrhizinate in vitro significantly reduces the expression of HSC activation markers and effectively alleviates the formation of hepatic fibrosis scars in a rat model of liver fibrosis [77]. ZFP36 ring finger protein (ZFP36/TTP) promotes ferroptosis in HSCs and may serve as a potential new target for the treatment of liver fibrosis [78]. Although some articles do not specifically describe SLC7A11-related ferroptosis, they document how promoting ferroptosis in HSCs can effectively improve liver fibrosis. The review by Tang et al. [79] discusses the intricate association between ferroptosis in HSCs and the pathogenesis of liver fibrosis. Targeting SLC7A11 to induce HSCs ferroptosis can ameliorate liver fibrosis. Therefore, downregulating SLC7A11 expression or reducing its activity in HSCs can induce ferroptosis.

#### Inhibiting SLC7A11 transcription to promote HSC ferroptosis and improve liver fibrosis

SLC7A11 can promote ferroptosis in HSCs and ameliorate liver fibrosis. Inhibiting sorafenib-induced ferroptosis may downregulate SLC7A11 transcription by reducing HIF-α levels. Thus, sorafenib can be used to reduce liver fibrosis [45]. Baicalin, an active ingredient in the traditional Chinese medicine Baicalein, decreases p53 activation by downregulating the suppressor of cytokine signaling 1 (an essential signal transduction regulator protein that is crucial for immune modulation, inflammation regulation, and immunity), which reduces SLC7A11 transcription and improved liver fibrosis [80]. Ginsenoside Rh2 (GRh2) negatively regulates SLC7A11 and promotes HSC ferroptosis by enhancing interferon regulatory factor 1 (IRF-1) activity [81]. In summary, ferroptosis in HSCs can be promoted by regulating the transcription factors upstream of SLC7A11 to reduce SLC7A11 transcription.

#### Other pathways that promote HSC ferroptosis and reduce fibrosis by affecting SLC7A11

Downregulation of SLC7A11 induces HSC ferroptosis, accompanied by an improvement in liver fibrosis through various mechanisms [48, 82-85]. Danshensu downregulates the expression of SLC7A11 in LPS-induced activated HSCs, promoting ferroptosis leading to reduced fibrosis [86]. TRIM26 can attenuate SLC7A11 activity and promote ferroptosis in HSCs through SLC7A11 ubiquitination, thereby reducing liver fibrosis [50]. Ginsenoside Rb1 can upregulate BECN1 and reduce fibrosis through its interaction with SLC7A11, resulting in SLC7A11 downregulation and promotion of ferroptosis in HSCs [54]. These findings suggest that downregulating SLC7A11 to promote HSC ferroptosis could be used as a therapeutic approach for treating liver fibrosis.

### The Diverse roles of SLC7A11 in hepatic fibrosis: mechanisms and interactions

#### SLC7A11 upregulation in hepatocytes protects against ferroptosis and reduces fibrosis

SLC7A11 is essential for preventing ferroptosis in hepatocytes [87], and SLC7A11 upregulation in hepatocytes, which confers protection against ferroptosis, can ameliorate hepatocyte injury and reduce fibrosis [74, 88, 89]. Antituberculosis drugs may induce ferroptosis in hepatocytes through the HIF-1α/SLC7A11/GPX pathway, leading to liver fibrosis [46]. In nonalcoholic steatohepatitis (NASH) models, the downregulation or absence of AGER1 decreased SLC7A11 expression, thereby inducing hepatocyte injury and exacerbating liver fibrosis [90]. SLC7A11 upregulation may ameliorate fibrosis at the macroscopic level by inhibiting ferroptosis in hepatocytes [91, 92]. These findings indicate that SLC7A11 upregulation prevents hepatic fibrosis by suppressing ferroptosis in hepatocytes.

#### Effect of SLC7A11 on the progression of fibrosis mediated by the interaction between different cells in liver tissue

SLC7A11 influences liver fibrosis through several mechanisms, presumably because of its diverse functions in different cell types. The intricate interplay between SLC7A11 and various liver tissue cells contributes to the complexity of liver fibrosis. This interplay can be summarized in three situations:

(1) *Synergistic effect in various cell types*: SLC7A11 exerts a “synergistic” or “combined” effect in various cell types. Specifically, SLC7A11 upregulation in hepatocytes initially affects liver cells and influences HSCs via the extracellular pathway, thereby attenuating HSC activation and protecting against liver fibrosis. The SLC7A11 content of liver tissue is elevated in patients with NASH and the SLC7A11 level is directly associated with the severity of liver damage [44]. In a mouse NASH model, liver fibrosis was markedly more pronounced in SLC7A11 knockout mice than in their wild-type counterparts under identical conditions, whereas SLC7A11 overexpression in mouse hepatocytes ameliorated liver fibrosis. SLC7A11 activates the 5′ adenosine monophosphate-activated protein kinase pathway by α-ketoglutarate and prolyl hydroxylase regulation and reduced ROS accumulation. This activation leads to increased mitophagy and decresed NOD-like recptor protein 3(NLPR3) inflammasome expression in liver tissue, ultimately resulting in reduced IL-1β accumulation in the liver. IL-1β can stimulate the inflammatory response in the liver and activate HSCs by recruiting myeloid cells, promoting collagen production and the development of liver fibrosis [93]. Thus, SLC7A11 overexpression in hepatocytes protects against the development of liver fibrosis by regulating hepatocytes, inhibiting inflammatory factor production, and reducing activated HSCs. The involvement of SLC7A11 in liver fibrosis extends beyond its effects on ferroptosis.
(2) *Antagonistic effects of SLC7A11 in hepatocytes and HSCs*: SLC7A11 upregulation in hepatocytes or HSCs can reduce ferroptosis in both cell types. SLC7A11 exerts its effects on both hepatocytes and HSCs simultaneously. In terms of limiting fibrosis, SLC7A11 upregulation in hepatocytes has a beneficial effect, whereas SLC7A11 upregulation in HSCs has a detrimental effect. Therefore, this phenomenon can be summarized as an “antagonistic” effect. Targeting SLC7A11 in the treatment of chronic liver injury fails to alleviate liver fibrosis and exacerbates hepatic damage, increasing markers associated with liver fibrosis. However, large doses of erastin, which induces ferroptosis in primary HSC cultures, do not decrease the viability of primary hepatocyte cultures [71]. This finding suggests that MF-HSCs have an increased sensitivity to ferroptosis inducers in settings of acute liver injury. This is supported by evidence that in mice, drugs targeting SLC7A11-induced ferroptosis in HSCs in a carbon tetrachloride (CCl_4_)-induced liver injury model do not affect the functionality of normal hepatocytes and macrophages. Furthermore, TGF-β may be a crucial function in the pathogenesis of liver fibrosis through epithelial-mesenchymal transition (EMT) based on a chronic liver injury model [71]. EMT increases the susceptibility of cultured hepatocytes to ferroptosis and dependence on xCT [62, 71, 94]. SLC7A11 is upregulated to transport increased cysteine to supplement GSH, maintain hepatocyte activity, and remove ROS, making hepatocytes susceptible to proferroptotic factors that target SLC7A11. Therefore, treatments that target SLC7A11 may affect normal hepatocytes. In summary, the efficacy of inhibiting SLC7A11 alone may be limited in ameliorating fibrosis in chronic liver injury models because of the limited difference in SLC7A11 dependence and ferroptosis sensitivity between hepatocytes and MF-HSCs. Moreover, SLC7A11 overexpression could potentially safeguard against the progression of liver fibrosis by suppressing the inflammatory response and reducing HSC activation.
(3) *Effects of SLC7A11 in inflammatory cells*: Limited evidence suggests that SLC7A11 may exert a direct or indirect influence on inflammatory cells within the liver, thereby influencing liver fibrosis progression through direct or indirect means (such as by affecting HSCs). SLC7A11-associated ferroptosis has been observed in bone marrow-derived macrophages [95]. GRh2 also reduces macrophage recruitment to the liver and regulates the concentrations of inflammatory factors in the liver [81]. Lv et al. [44] demonstrated a close association between SLC7A11 and IL-1β levels in hepatic tissue, with IL-1β being closely linked to liver fibrosis. Pang et al. [96] found that downregulating the IRF-1/SLC7A11 axis in inflammatory macrophages can promote ferroptosis in these cells and improve liver fibrosis. The xCT transporter in peripheral blood monocytes of individuals with advanced cirrhosis markedly affects the normal immune function of monocytes [97].

### Dual Role of SLC7A11 in liver fibrosis: cell-specific effects and future directions

**Figure 3.**
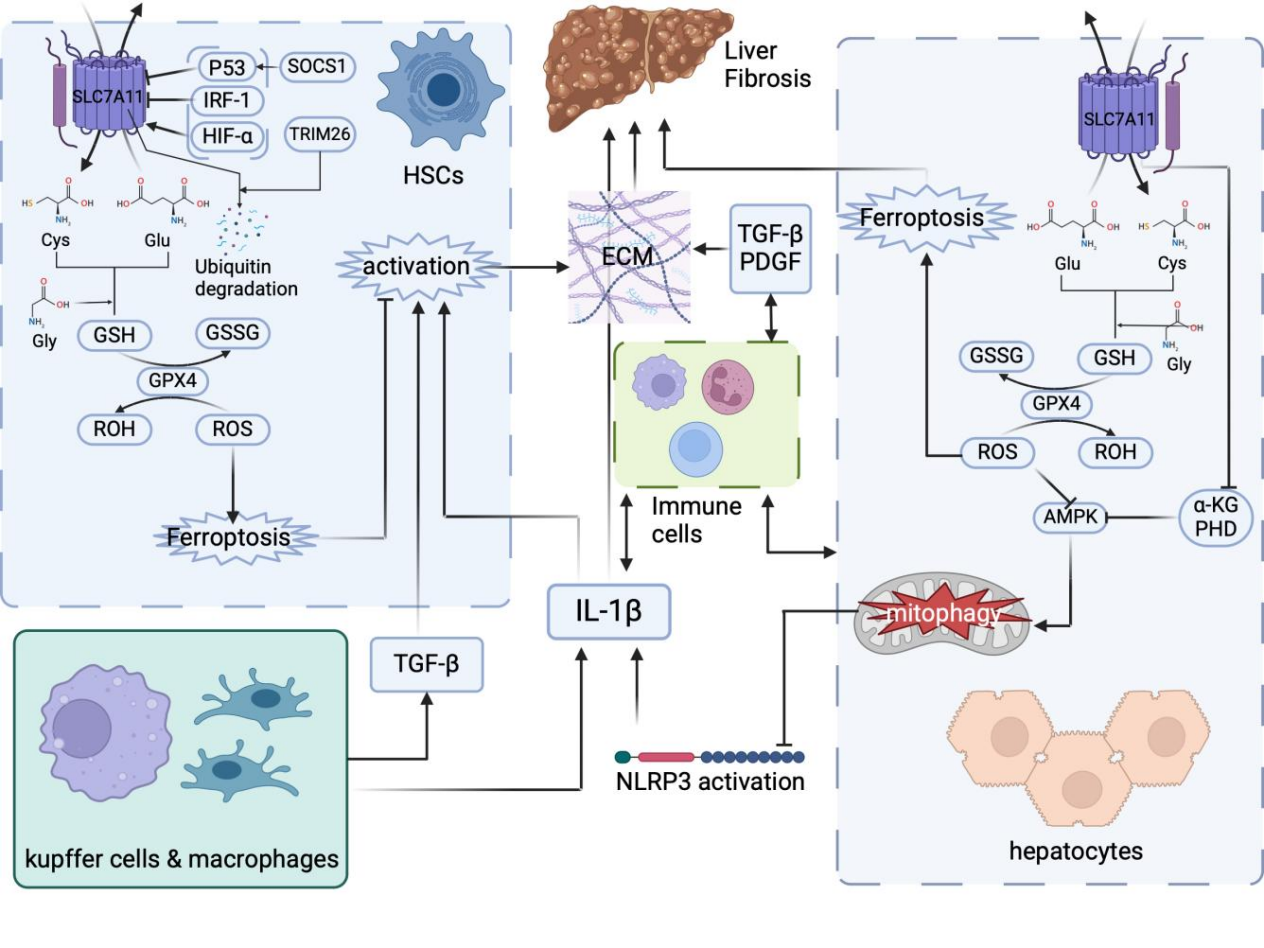
**Role of SLC7A11 in liver fibrosis**. In hepatic stellate cells (HSCs), SLC7A11 is negatively regulated by upstream factors such as suppressor of cytokine signaling 1 (SOCS1)/p53 and IRF-1 and positively regulated by HIF-1α. SLC7A11 can also undergo TRIM26-mediated ubiquitination and degradation. Downstream, SLC7A11 primarily functions through the classic pathway by transporting cystine, promoting GSH synthesis, and reducing ROS, thereby protecting against ferroptosis. However, ferroptosis in HSCs inhibits their activation and reduces excessive extracellular matrix (ECM) production, ultimately exerting a protective effect against liver fibrosis. Consequently, SLC7A11 plays a profibrotic role in this pathway. SLC7A11 has complicated effects on hepatocytes. SLC7A11 inhibits ferroptosis in hepatocytes, thereby providing a protective effect against liver fibrosis. SLC7A11 downregulates α-ketoglutarate (α-KG), prolyl hydroxylase enzymes (PHD), and ROS, alleviating their inhibitory effects on 5′ adenosine monophosphate-activated protein kinase (AMPK). This facilitates AMPK-mediated mitophagy, reduces NOD-like receptor protein 3 (NLRP3) inflammasome activation, and decreases interleukin-1β (IL-1β) production. IL-1β can promote liver fibrosis through pathways, including HSC activation. Therefore, in this pathway, SLC7A11 exerts a protective effect against liver fibrosis. Additionally, Kupffer cells, liver-recruited macrophages, and other immune cells can influence liver fibrosis through transforming growth factor beta (TGF-β), IL-1β-dependent pathways, or other unknown mechanisms.

In summary, the inconsistent effects of SLC7A11 on different liver cells suggest that it does not exert a singular effect on liver fibrosis. SLC7A11 upregulation in hepatocytes can reduce liver fibrosis directly or by inhibiting HSC activation through inflammatory factors, thereby having a beneficial effect. Conversely, SLC7A11 downregulation in HSCs to induce ferroptosis also reduces liver fibrosis. Current understanding of the overall effect of SLC7A11 on hepatocytes, inflammatory cells, and HSCs in the liver tissue is limited, particularly the understanding of the role of inflammatory cells. The role of SLC7A11 in liver fibrosis is summarized in Figure 3. Future research should investigate the presence of SLC7A11 in specific cell types and assess its influence on other cell types to investigate the interactions between different cell populations within the liver tissue.

### Drug development possibilities targeting SLC7A11 for treating liver fibrosis

The development of drugs aimed at targeting SLC7A11 for treating liver fibrosis is in its preliminary stages. Although various drugs targeting SLC7A11 have been identified, research on drug side effects and absorption, distribution, metabolism, and excretion is limited. The research and development of these drugs is primarily theoretical, with a lack of focus on their practical applications or clinical experiments.

Du et al. [71] highlighted the importance of inducing HSC death by targeting SLC7A11. However, further research is needed on the potential side effects drugs that target SLC7A11, and the development of treatments that specifically target HSCs while sparing normal liver cells and macrophages. A drug delivery system (DDS) is a technical system that comprehensively regulates the distribution of drugs in the organism in terms of space, time, and dose. The goal is to deliver the right amount of drug to the right place at the right time, to increase the utilization efficiency of drugs, improve efficacy, reduce costs, and reduce side effects. Drug targeting can be achieved through drug delivery systems, including nanoformulations such as liposomes. Targeted liposomes may be a feasible approach, and drug efficacy may be enhanced through improved targeting of MF-HSCs by incorporating specific MF-HSC markers onto the liposomal surface. A review by Schuppan et al. [98] reported that inactivated liposomes and nanoparticles can be preferentially engulfed by HSCs, serving as carriers for small molecules and providing a means of targeting HSCs. Wang et al. [99] and Pei et al. [100] reviewed recent advances in various nanoformulations, including baicalin liposomes [99], which enhance drug absorption and targeting and minimize uptake by off-target cells in other organs, thereby reducing the side effects.

In addition to nanoparticle formulations, Schuppan et al. [98] reviewed other potential targeting strategies. Agents can be covalently or non-covalently attached to carriers that are modified with ligands designed to bind specifically to receptors on activated HSCs. Many siRNA and antisense oligonucleotides have been made to be highly liver specific, and antisense single-stranded DNA/RNA oligonucleotides (ASOs) can be taken up by specific cells, including macrophages, hepatocytes and HSCs [101].

Several agents that may target SLC7A11 in HSCs have been proposed to treat liver fibrosis. Liu et al. [102] successfully developed aggregation-induced emission carbon nitride-based nanoparticles. The incorporation of vitamin A units enabled targeted delivery to HSCs while simultaneously downregulating SLC7A11 through HIF-1α inhibition. Inducing ferroptosis can potentially ameliorate fibrosis by targeting activated HSCs. Tran et al. [103] developed sorafenib-loaded silica containing REDOX nanoparticles as an oral drug for the treatment of liver fibrosis. The use of nanoparticles significantly improved the antifibrotic efficacy, increased liver distribution, and reduced gastrointestinal toxic side effects of sorafenib. The development of SLC7A11-targeting drugs for the treatment of liver fibrosis treatment is an exciting avenue for future research.

### Future perspectives on using SLC7A11-targeting drugs to treat liver fibrosis

Targeting SLC7A11 to induce ferroptosis in HSCs and mitigate the accumulation of fibrosis-related substances in the liver can be used to address liver fibrosis. However, several challenges remain unresolved. A primary concern is that ferroptosis-inducing drugs have severe adverse effects. Targeting the downregulation of SLC7A11 can induce ferroptosis in HSCs, thereby improving fibrosis, while also inducing ferroptosis in hepatocytes, which can exacerbate fibrosis. If small molecules (such as erastin) are used as delivery agents, their potential cytotoxicity, especially hepatotoxicity and nephrotoxicity, should not be overlooked. Additionally, because SLC7A11 is ubiquitously expressed in cells throughout the body, it is likely to influence the metabolism of other cell types, particularly events related to oxidative stress and ferroptosis, potentially leading to numerous adverse effects. The adverse effects of drugs targeting SLC7A11 are serious but can sometimes be reduced by using DDS targeting HSCs. Clinical trials of drugs targeting SLC7A11 also face major challenges. Efficacy demonstrated in rodent models may not necessarily translate to success in the complex multicellular environment of the human body and could lead to side effects in other organs and cells. The standards for preclinical drug trials need further refinement [98]. The review by Zhao et al. [104] discusses several targeted antifibrotic drugs currently undergoing clinical trials. Furthermore, SLC7A11 may influence fibrosis through an alternative mitophagy pathway. Future research should develop drugs targeting HSCs to trigger ferroptosis within these cells and investigate the mechanism by which SLC7A11 affects fibrosis via alternate (nonferroptotic) pathways.

## Role of SLC7A11 in PF

### Introduction of PF

The end-stage lung pathology of interstitial lung disease is marked by fibroblast proliferation, substantial ECM deposition, and structural destruction of lung tissue, resulting in PF [105]. The diseases that result in PF often manifest in the lung interstitium and are collectively referred to as interstitial lung diseases. The most prevalent and progressive form of PF is IPF, which causes severe disease and has a poor prognosis [4]. Other forms of PF can occur owing to autoimmune disease or exposure to toxic substances.

The pathogenesis of PF, similar to that of other fibrotic diseases, involves excessive ECM production in response to inflammatory stimuli, leading to structural and functional alterations in the lung. However, research on PF has primarily focused on IPF, and the exact pathogenesis of PF remains unknown. A combination of genetic factors and accelerated cellular senescence, primarily affecting alveolar epithelial type 2 cells(AT2), and complex interactions among alveolar epithelial, interstitial, and ECM components play a role in the progression of IPF [105]. Repeated microinsults leading to AT2 depletion [106], as well as myofibroblasts, may be the underlying mechanism for PF [107]. During normal healing, myofibroblasts undergo apoptosis and re-epithelialization. However, in IPF, myofibroblasts are unable to complete these processes and continue to generate excessive ECM, contributing to the progression of IPF [105]. Myofibroblasts are resistant to apoptosis during chronic inflammation, leading to aberrant wound healing and excessive ECM production, ultimately resulting in PF [108]. The inflammatory response is also crucial in PF onset and progression, and the innate and adaptive immune systems influence the development and progression of PF [109]. Different immune cell populations may have different effects on PF [105].

### Mechanism of PF

Many pathways are involved in PF, including the TGF-β, Wnt/β-catenin, hedgehog, Notch, and fibroblast growth factor signaling pathways. TGF-β can influence the synthesis of ECM and inhibit its degradation through the SMAD signaling pathway. Additionally, it promotes the progression of fibrosis by inducing the transformation of fibroblasts into myofibroblasts [110-113]. The canonical Wnt signaling pathway inhibits the phosphorylation of intracellular β-catenin, allowing it to translocate into the nucleus, where it activates the transcription factors TCF/LEF and modulates the expression of various genes [114, 115]. The Wnt/β-catenin pathway may promote the progression of PF by facilitating the conversion of fibroblasts to myofibroblasts, activating AEC II cells to increase IL-1β production, and stimulating inflammation and profibrotic responses [116, 117]. In addition, several proinflammatory factors and cellular markers are involved in this pathological process, including connective tissue growth factor (CTGF), PDGF, insulin growth factor, interleukin 4, IL-13, interferon-γ (IFN-γ), IL-1β, tumor necrosis factor-α (TNF-α), IL-17, oncostatin M, and IL-10 [113]. IL-1β and TNF-α may promote PF by enhancing the production of TGF-β [117, 118], whereas PDGF is a potent mitogen and chemotactic factor for lung myofibroblasts [119].

### SLC7A11 improves PF through ferroptosis-dependent pathway

Research on the association between SLC7A11 and PF is limited; however, SLC7A11 downregulation is associated with PF, whereas SLC7A11 upregulation is associated with improvements in the clinical manifestations and histological structure of PF. SLC7A11 may affect PF by modulating ferroptosis in various cell types. A bleomycin-induced PF model has demonstrated a decrease in SLC7A11 level in mice with bleomycin-induced PF [120]. Silicon dioxide (SiO_2_) accumulates within lung macrophages and induces ferroptosis in alveolar macrophages [121-123]. Ma et al. [124] proposed that SiO_2_ may enhance ferroptosis in alveolar macrophages and facilitate silicosis progression by downregulating SLC7A11. Molybdenum and cadmium may promote PF progression by downregulating SLC7A11 to induce ferroptosis and activate caveolin-1 (CAV-1) and the Wnt/β-catenin pathway [125]. SLC7A11 upregulation induced by certain drugs can inhibit ferroptosis in the lung tissue and reduce fibrosis [120, 126]. These findings suggest that SLC7A11 downregulation and ferroptosis are involved in PF.

Although the specific molecular mechanisms remain poorly defined, by inducing lung fibroblast apoptosis, ferroptosis is crucial in PF progression [127]. Therefore, the effect of SLC7A11 on ferroptosis in lung fibroblasts warrants further investigation. SLC7A11 suppresses ECM deposition in PF by inhibiting ferroptosis in fibroblasts, thereby reversing their senescent phenotype and attenuating the production of fibrosis-related markers [128]. Treatment with tuberostemonine alleviates lung fibrosis in mice, as it interferes with TGF-β1-induced fibroblast proliferation. Additionally, it reverses erastin-induced ferroptosis by upregulating SLC7A11 expression and inhibiting ferroptosis in fibroblasts [120]. These findings suggest that SLC7A11 upregulation can inhibit ferroptosis in lung fibroblasts but reduce ECM deposition during PF. Therefore, this specific intrinsic mechanism may be associated with myofibroblasts, and SLC7A11 may be involved in modulating the complex interactions between lung epithelial cells, macrophages, and fibroblasts during fibrosis.

### SLC7A11 improves PF through ferroptosis-independent pathway

The effect of SLC7A11 on PF progression is likely mediated by nonferroptotic pathways. SLC7A11 exhibits overlapping mechanisms in ferroptosis inhibition and EMT promotion [129]. Furthermore, ferroptosis and EMT may interact, and SLC7A11 downregulation may exacerbate fibrosis [129]. Although the relationship between EMT and fibrosis remains unclear, SLC7A11 downregulation is associated with exacerbated PF, and the underlying mechanism likely extends beyond merely inhibiting ferroptosis.

### Role and Potential of SLC7A11 in PF

**Figure 4.**
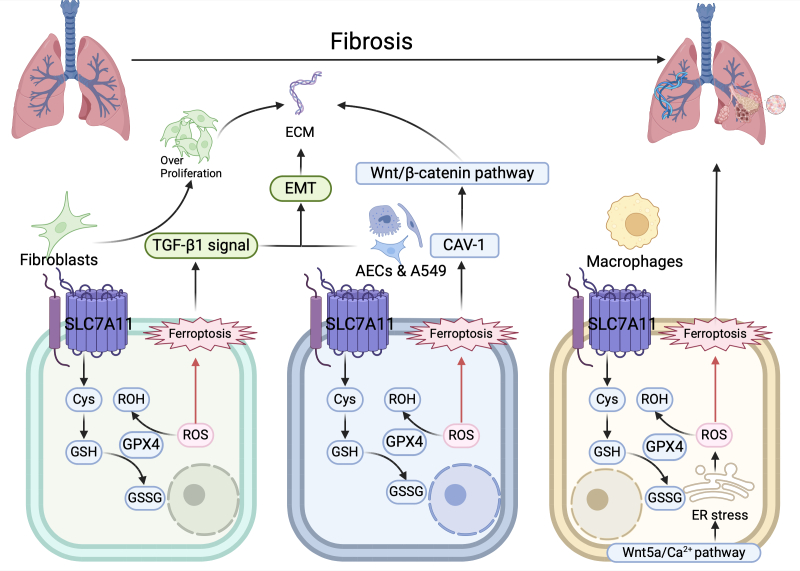
**Role of SLC7A11 in pulmonary fibrosis**. In lung fibroblasts, SLC7A11 counteracts ferroptosis, thereby reducing TGF-β signaling pathway activation. TGF-β signaling pathway activation promotes excessive fibroblast proliferation and ECM accumulation, leading to fibrosis. Similarly, in lung epithelial cells, SLC7A11 inhibits ferroptosis and downregulates caveolin-1 (CAV-1), which reduces Wnt/β-catenin pathway activation, thereby decreasing ECM production. TGF-β signaling pathway activation can induce epithelial-mesenchymal transition (EMT) in lung epithelial cells, leading to excessive ECM production. Wnt5a/calcium (Wnt5a/Ca^2+^) signaling pathway activation can induce endoplasmic reticulum stress in lung macrophages, resulting in excessive ROS production. SLC7A11 reduces ROS levels, thereby counteracting ferroptosis. Macrophage ferroptosis can contribute to the development of pulmonary fibrosis. Overall, SLC7A11 combats pulmonary fibrosis through multiple pathways, acting on lung fibroblasts, lung epithelial cells, and macrophages.

In summary, evidence on the role of SLC7A11 in PF is scarce relative to the evidence of its role in liver fibrosis; however, SLC7A11 downregulation in the lung is associated with PF (Fig. 4). This mechanism may be partially attributable to SLC7A11 upregulation inhibiting ferroptosis in alveolar epithelial cells and fibroblasts. Further research is needed to investigate the involvement of SLC7A11 in the interaction of various cells within lung tissue. Targeting the SLC7A11/GPX4 axis is a potential therapeutic target for treating PF [128]. Further research is required to investigate the mechanisms by which SLC7A11 influences PF.

### The Role of SLC7A11 in renal fibrosis: mechanisms and therapeutic implications

Chronic kidney disease (CKD) is a progressive condition characterized by changes in kidney structure and function, often advancing to kidney failure [130, 131]. Renal interstitial fibrosis (RIF), marked by glomerular sclerosis and tubular fibrosis, is a defining feature of CKD [132, 133]. The primary feature of RIF is the pathological deposition of ECM, resulting in an imbalance in ECM production and elimination, leading to a deterioration in kidney structure and function [134]. Research on the mechanisms underlying RIF is limited, but myofibroblasts play a central role in the progression of renal fibrosis [135]. Stimulation with profibrotic factors such as TGF-β1 transforms kidney-resident fibroblasts into myofibroblasts and actively contributes to ECM deposition. Other kidney-resident cells, such as renal tubular epithelial cells, endothelial cells, fibroblasts, Sertoli cells, mesangial cells, and macrophages, are also involved in ECM synthesis [136].

Excessive ECM production also promotes fibrosis by regulating multiple common factors, including TGF-β1 [137]. In contrast, the TGF-β family member, bone morphogenetic protein 7 (BMP-7), exerts an antifibrotic role in renal fibrosis [138]. CTGF is an additional factor that influences fibrosis by augmenting the signaling potency of TGF-β and diminishing the antifibrotic effect of BMP-7 [139]. Cytokines, such as IL-1β and IL-6, are also involved in ECM production [140]. However, IFN-γ can attenuate the deposition of ECM [141]. Currently, effective pharmacological treatments for managing renal fibrosis are limited [142, 143]. In Western countries, patients with kidney failure commonly receive kidney transplantation or dialysis as the primary treatment to restore functionality. Although traditional Chinese medicines are sometimes used for treatment of renal fibrosis in China, dialysis and kidney transplantation are the mainstay therapies. The review by Nastase et al. [136] provides further information on renal fibrosis.

Recent research suggests that SLC7A11 plays a protective role in renal fibrosis, particularly through the SLC7A11/GPX4/GSH axis, which may inhibit ferroptosis in renal tubular epithelial cells and podocytes, thereby preventing kidney damage and reducing fibrosis [144]. Various renal fibrosis models have consistently demonstrated SLC7A11 downregulation in renal fibrosis [35, 145-153]. Exposure to copper induces kidney fibrosis in chickens, accompanied by SLC7A11 downregulation and ferroptosis [154]. Furthermore, human proximal tubular epithelial cells [150, 152, 153], mouse renal tubular epithelial cells (TECs) [145, 147, 148], and mouse renal podocytes [149, 151] also underwent ferroptosis accompanied by SLC7A11 downregulation. Correspondingly, SLC7A11 is upregulated by drugs that ameliorate renal fibrosis. Fer-1 inhibits ferroptosis and improves fibrosis in renal TECs by upregulating SLC7A11 and GPX4 expression [147]. Certain traditional Chinese medicine treatments can upregulate SLC7A11 expression and inhibit ferroptosis, leading to reduced renal fibrosis [146, 148-153]. These studies demonstrate that inhibiting ferroptosis and upregulating SLC7A11 can decrease renal fibrosis. Research has demonstrated that EZH2 can epigenetically regulate and downregulate SLC7A11 expression, leading to renal fibrosis [35]. However, the effect of drugs that target EZH2 on SLC7A11 expression remains unclear.

**Figure 5.**
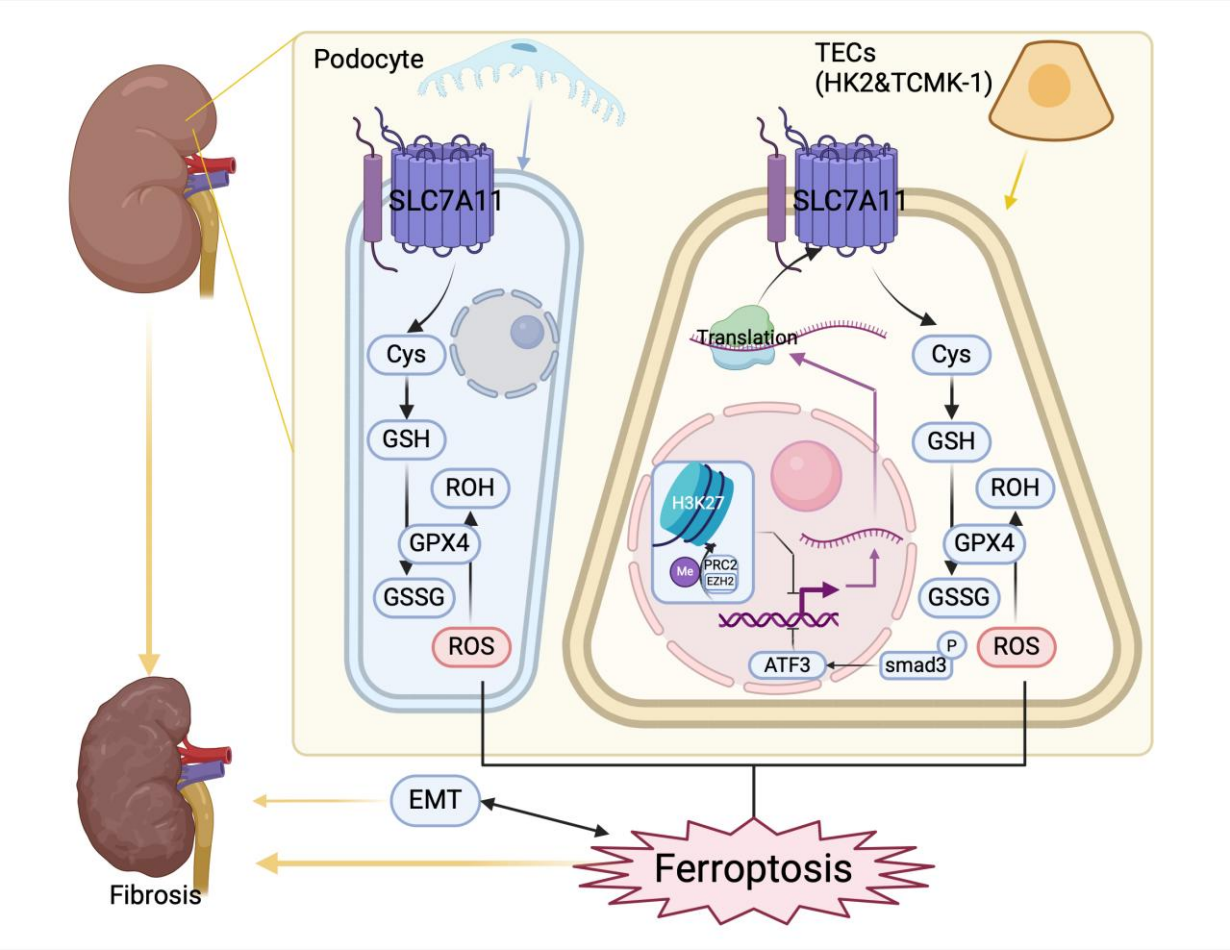
**Role of SLC7A11 in kidney fibrosis**. In renal tubular epithelial cells, SLC7A11 is epigenetically regulated upstream by EZH2, a component of PRC2. EZH2 inhibits *SLC7A11* gene transcription by promoting H3K27 methylation. Concurrently, phosphorylated SMAD family member 3 (SMAD3) suppresses SLC7A11 gene transcription by activating ATF3. Downstream, SLC7A11 reduces ROS through the GSH/GPX4 pathway, counteracting ferroptosis. SLC7A11 also reduces ROS levels to inhibit ferroptosis in podocytes. Overall, SLC7A11 alleviates renal fibrosis by counteracting ferroptosis. SLC7A11 upregulation is associated with reduced ferroptosis and inhibition of podocyte EMT.

Several studies have concentrated on targeting ferroptosis and several potential underlying mechanisms through which SLC7A11 mitigates renal fibrosis have been proposed. The EMT and ferroptosis pathways intersect [129, 155], and SLC7A11 upregulation is accompanied by EMT attenuation in renal tubular cells [151]. Despite the lack of consensus regarding the role of EMT in renal fibrosis, further research is warranted to assess whether SLC7A11 can improve fibrosis by improving EMT.

In mice with arsenic- and polystyrene nanoplastic-induced kidney fibrosis, ferroptosis occurs concomitantly with an imbalance in mitochondrial dynamics, resulting in increased mitochondrial ROS and SLC7A11 downregulation [156]. The presence of SLC7A11 prevents excessive ROS accumulation, thereby directly affecting ferroptosis. Therefore, SLC7A11 may affect renal fibrosis through both its effects on mitochondrial dynamics and ferroptosis. Further studies are warranted to explore the role of SLC7A11 in mitochondrial dynamic imbalance and to investigate the contributions of mitophagy and ferroptosis to the progression of renal fibrosis.

The role of SLC7A11 in kidney fibrosis is summarized in Figure 5. SLC7A11 upregulation is accompanied by ferroptosis inhibition and an improvement in renal fibrosis. However, the role of SLC7A11 in myofibroblasts, which are a crucial source of ECM during renal fibrosis, remains unclear. Additionally, SLC7A11 may affect renal fibrosis through other mechanisms, such as mitophagy. Furthermore, cells affected by SLC7A11 may interact. Further research may provide a clearer understanding of the relationship between SLC7A11 and renal fibrosis and potentially lead to the development of targeted therapies.

### Role of SLC7A11 in myocardial fibrosis

Myocardial fibrosis is characterized by the development of the myocardial interstitium resulting from the deposition of ECM proteins, which is a shared pathophysiological characteristic of different myocardial diseases. Various cardiac pathological changes may accompany myocardial fibrosis [157]. Microscopically, myocardial fibrosis is categorized into two categories based on the pattern of fiber deposition: replacement or reparative and reactive fibrosis (including interstitial and perivascular fibrosis) [158]. The mammalian heart cannot regenerate, resulting in scar tissue formation as a replacement for dead myocardial cells. Myocardial infarction is a classic example of reparative fibrosis in which the death of multiple cardiomyocytes stimulates an inflammatory response that activates reparative myofibroblasts, leading to scar formation [159]. Reactive fibrosis primarily represents a pathological condition characterized by persistent ECM accumulation in the absence of myocardial cell loss. Common etiologies include pressure or volume overload [160]. Cardiac fibrosis is frequently accompanied by concurrent pathological processes collectively affecting the heart. Therefore, the extent of myocardial fibrosis is commonly used as an indicator of ventricular remodeling and myocardial hypertrophy.

Myocardial fibrosis is also closely associated with cardiac fibroblast (CF) activation and excessive ECM production [161]. Cardiac fibrosis is driven primarily by myofibroblasts, which are the predominant cellular contributors [162]. The injured microenvironment can provoke the differentiation of CFs into myofibroblasts when the structural integrity of the myocardium is disrupted [163]. The exocrine system, Ang II, interleukin 6 (IL-6), and other factors participate in cellular interactions and influence fibrosis. More detailed information is provided in the reviews by Frangogiannis [160], Maruyama and Imanaka-Yoshida [161], and Liu et al. [162].

SLC7A11 may reduce the progression of cardiac fibrosis by suppressing ferroptosis in myocardial parenchymal cells, primarily in cardiomyocytes. In vivo and in vitro studies have demonstrated ferroptosis and SLC7A11 downregulation in mouse cardiomyocytes [47, 164-168]. Myocardial fibrosis, accompanied by ferroptosis and SLC7A11 downregulation, has also been observed in canine models of atrial fibrillation and rat H9C2 cells [169]. Additionally, xCT knockout mice exhibit increased fibrosis under same triggers [47], providing strong evidence that SLC7A11 plays a protective role in preventing myocardial fibrosis. Drugs that reduce myocardial fibrosis are associated with increased SLC7A11 expression within cardiomyocytes and reduced ferroptosis [169-171]. Fer-1 directly upregulates SLC7A11 to reduce cardiac ferroptosis and fibrosis [167, 169]. Sirtuin 1 (Sirt1)/p53 pathway activation by resveratrol mitigates SLC7A11 depletion, inhibits ferroptosis, and reduces cardiac fibrosis [171].

The findings of fibrosis replacement or repair models are generally consistent. In a murine model of ischemia-reperfusion-induced myocardial fibrosis, myocardial fibrosis was concomitant with ferroptosis and SLC7A11 downregulation. Dexmedetomidine treatment inhibits ferroptosis and upregulates SLC7A11, thereby reducing myocardial fibrosis [172, 173]. In a post-myocardial infarction fibrosis model, SLC7A11 upregulation was accompanied by reduced fibrosis in rats with heart failure [174]. These findings suggest that SLC7A11 may improve myocardial fibrosis by inhibiting ferroptosis in cardiomyocytes.

**Figure 6.**
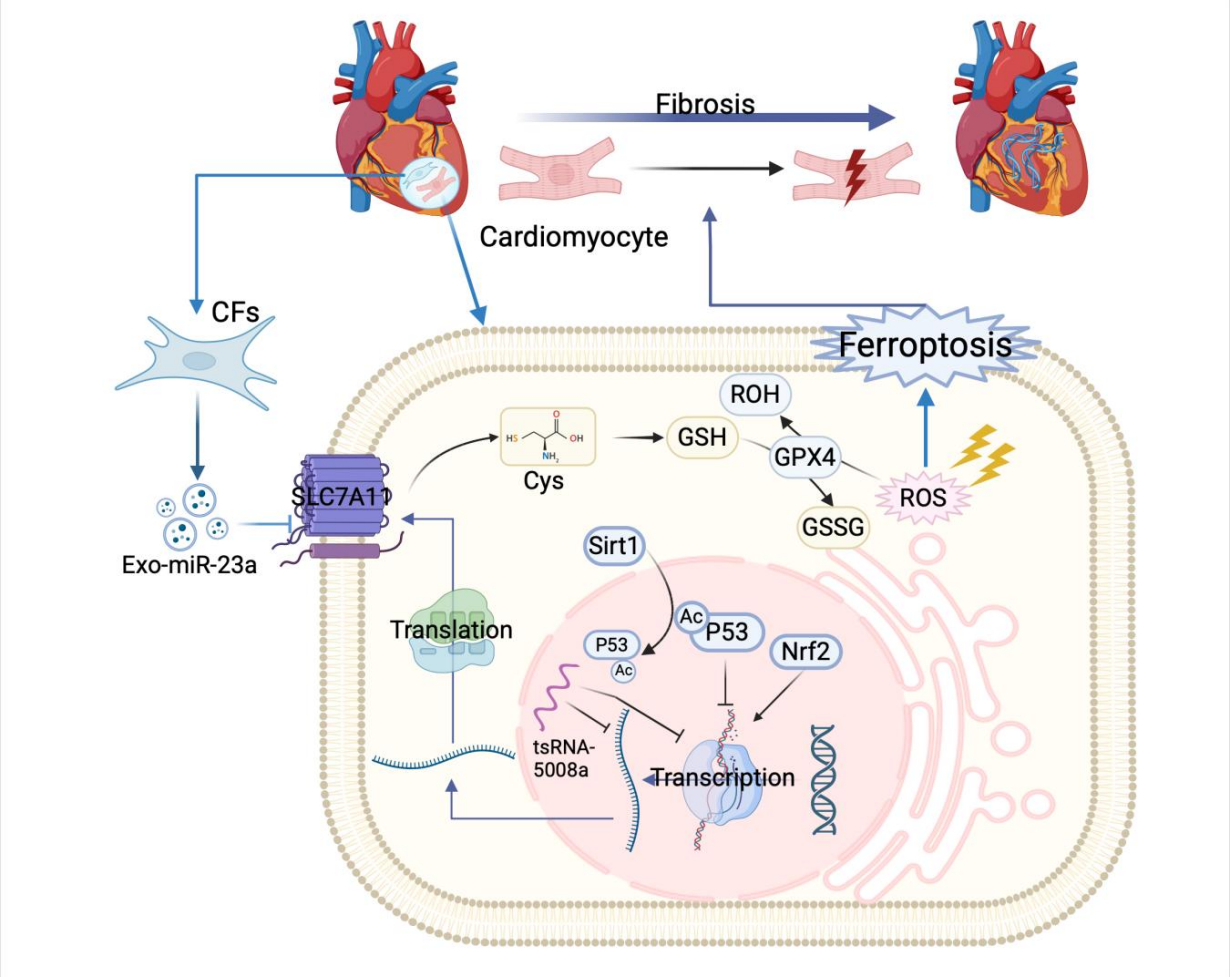
**Role of SLC7A11 in myocardial fibrosis**. In cardiomyocytes, SLC7A11 is negatively regulated upstream by acetylated p53, whereas sirtuin 1 (Sirt1) can deacetylate p53, thereby removing its inhibitory effect on SLC7A11 transcription. Additionally, tRNA-derived small RNAs-5008a (tsRNA-5008a) may negatively regulate SLC7A11 by inhibiting its gene transcription or reducing *SLC7A11* mRNA activity. *SLC7A11* is also a target gene of exosomal microRNA-23a (Exo-miR-23a) and can be negatively regulated by fibroblast-derived exosomes carrying Exo-miR-23a. Conversely, Nrf2 positively regulates SLC7A11 by promoting its transcription. Downstream, SLC7A11 counteracts ferroptosis by reducing ROS, thereby inhibiting cardiac fibrosis development.

The role of SLC7A11 in myocardial fibrosis is summarized in Figure 6. Few targeted studies have examined the relationship between SLC7A11 and cardiac fibrosis. Thus, the association between SLC7A11 and myocardial fibrosis is unclear [175]. Drug therapy with unspecified targets suggests SLC7A11 upregulation may improve cardiac fibrosis by mitigating ferroptosis. The precise mechanism by which SLC7A11 protects against myocardial fibrosis remains unclear, and further research is warranted. Nonetheless, SLC7A11 expression confers protection against myocardial fibrosis, making it a potential target for future therapeutic interventions. Therefore, future research should explore the effect of SLC7A11 on cardiomyocyte ferroptosis beyond conventional drug treatments with unclear targets and focus on investigating its specific role in fibrotic diseases by investigating the mechanisms underlying myocardial fibrosis. Considering the diverse effects of SLC7A11 in different cell types and their intricate interactions within cardiac tissue would lay a solid foundation for future drug development to treat myocardial fibrosis.

### Effects of SLC7A11 on pancreatic fibrosis

Similar to HSCs in the liver, pancreatic stellate cells (PSCs) are the primary contributors to pancreatic fibrosis. Normally functioning as vitamin A storage cells, PSCs can become activated and transform into myofibroblasts, secreting excessive collagen fibers under conditions like oxidative stress, which leads to fibrosis [176]. This process parallels that of HSCs in the liver. PSC activation is closely related to pancreatic inflammation and cytokine production. Macrophage-secreted factors such as PDGF, TGF-β, and TNF-α can promote PSC activation and ECM production [177]. Additionally, other cytokines, such as IL-1 and IL-6, also affect fibrosis by activating pancreatic PSCs [178].

Although SLC7A11 has not been demonstrated to be directly associated with pancreatic fibrosis, research on its relationship with pancreatic tumors and inflammation has revealed promising new avenues for fibrosis studies. SLC7A11 expression is upregulated in pancreatic ductal adenocarcinoma (PDAC) tissues, particularly in malignant proliferating cells [179]. Furthermore, cysteine depletion in tumor cells induces ferroptosis. Pancreatic acinar cell dedifferentiation is a hallmark of acute and chronic pancreatitis and is the initiating step in PDAC development. Pan et al. [180] found that in response to external stimuli, SLC7A11 is upregulated in acinar cells to evade ferroptosis, leading to acinar cell dedifferentiation and the progression of pancreatic neoplastic disease [180]. These findings suggest that SLC7A11 is a potential therapeutic target for PDAC.

Stable SLC7A11 knockdown in cancer-associated fibroblasts (CAFs) reduces tumor incidence, growth, and fibrosis [181]. These findings highlight the therapeutic promise of using gene silencing or nanomedicine techniques to inhibit SLC7A11 expression in PDAC tumors. The promotion of ferroptosis in CAFs suggests a potential approach for alleviating cancer-related fibrosis. Therefore, reducing SLC7A11 expression in PSCs and inducing ferroptosis may attenuate collagen production by PSCs and could potentially be used for the treatment of pancreatic fibrosis. This warrants further research. Future studies should focus on developing and evaluating strategies to inhibit the expression or function of SLC7A11 in PSCs and CAFs. This could be achieved through a range of approaches:

**Gene editing technologies**: CRISPR-Cas9 can be used to knock out the SLC7A11 gene, providing a permanent solution to eliminate its expression. However, challenges such as delivery specificity and off-target effects need to be addressed for therapeutic application.

**RNA interference**: Small interfering RNA (siRNA) or short hairpin RNA (shRNA) can be used to silence SLC7A11 expression. Advances in delivery systems, such as lipid nanoparticles, may improve the efficacy and specificity of this approach.

**Small molecule drugs**: Compounds such as erastin, which directly inhibit SLC7A11 activity, can induce ferroptosis in target cells. Further research is needed to identify more specific and less toxic small molecules for clinical use.

Additionally, a deeper understanding of the mechanisms by which SLC7A11 regulates ferroptosis is essential. The role of SLC7A11 in cystine uptake and glutathione synthesis is well-established, but its interactions with other cellular pathways and its precise effect on lipid peroxidation require further investigation. This knowledge will help in designing more effective and targeted therapies.

In particular, investigating how SLC7A11 expression is regulated in PSCs and how its inhibition affects their activation and collagen production will be crucial for developing treatments for pancreatic fibrosis. Similarly, understanding the role of SLC7A11 in pancreatic cancer progression and its potential as a target for combination therapy could lead to novel treatment strategies.

Overall, multidisciplinary approaches combining molecular biology, pharmacology, and clinical research are necessary to realize the therapeutic potential of targeting SLC7A11 in pancreatic fibrosis and pancreatic cancer.

### Effects of SLC7A11 on intestinal fibrosis and fibrosis in other organs

Inflammatory bowel disease (IBD), which includes ulcerative colitis (UC) and Crohn’s disease (CD), is a recurrent inflammatory disorder affecting the gastrointestinal tract. Intestinal fibrosis is a common and severe complication of CD and can lead to obstruction necessitating surgical intervention [182]. In UC, colonic fibrosis contributes to motility abnormalities [183]. During intestinal fibrosis, excessive ECM accumulation is driven primarily by intestinal mesenchymal cells—including fibroblasts, myofibroblasts, and smooth muscle cells—which secrete ECM [184]. After injury or inflammation, activated fibroblasts migrate to the injury site and proliferate, promoting ECM production. Additionally, pericytes, located around blood vessels and adjacent to endothelial cells, and EMT are involved in intestinal fibrosis [185, 186]. The inflammatory response which is mediated by various cytokines, is central to intestinal fibrosis [184]. TGF-β is engaged in intestinal fibrosis, enhancing myofibroblast migration and collagen production [187].

Currently, no treatments specifically target SLC7A11 to alleviate fibrosis in IBD. However, understanding the association between SLC7A11 and IBD may provide valuable insights. IBD is closely associated with impaired differentiation of intestinal epithelial cells [188]. Pan et al. [189] showed that vitronectin promotes ferroptosis of intestinal epithelial cells, leading to differentiation disorders and SLC7A11 and GPX4 downregulation. Electroacupuncture upregulated SLC7A11 expression in mice with IBD, affecting IBD through ferroptosis [188].

Additionally, STAT1-IRF-1-ACSL4 axis-dependent ferroptosis induction is a key factor in radiation-induced interstitial fibrosis [190]. In ischemia-reperfusion injury models, pharmacological ATF3 downregulation upregulates SLC7A11 expression and prevents ferroptosis, mitigating intestinal damage [191]. The protective effect of SLC7A11 on intestinal injury and its effect on IBD by preventing ferroptosis suggests that it may influence intestinal fibrosis by modulating ferroptosis.

To date, no independent anti-intestinal fibrosis drugs have been approved through clinical trials [184, 192]. IBD can lead to intestinal fibrosis, and current treatment options remain largely limited to surgical intervention. Therefore, the development of antifibrotic drugs for this condition is warranted. Preclinical studies targeting TGF-β as an antifibrotic approach are already underway [193]. However, the development of such drugs faces several challenges. As previously mentioned, one major issue is the difficulty of translating preclinical findings into clinical trials. Another challenge is the lack of validated clinical endpoints capable of accurately measuring fibrosis in the context of intestinal fibrosis [192]. A deeper understanding of the relationship between SLC7A11 and intestinal fibrosis could lay the foundation for its use as a therapeutic target.

**Figure 7.**
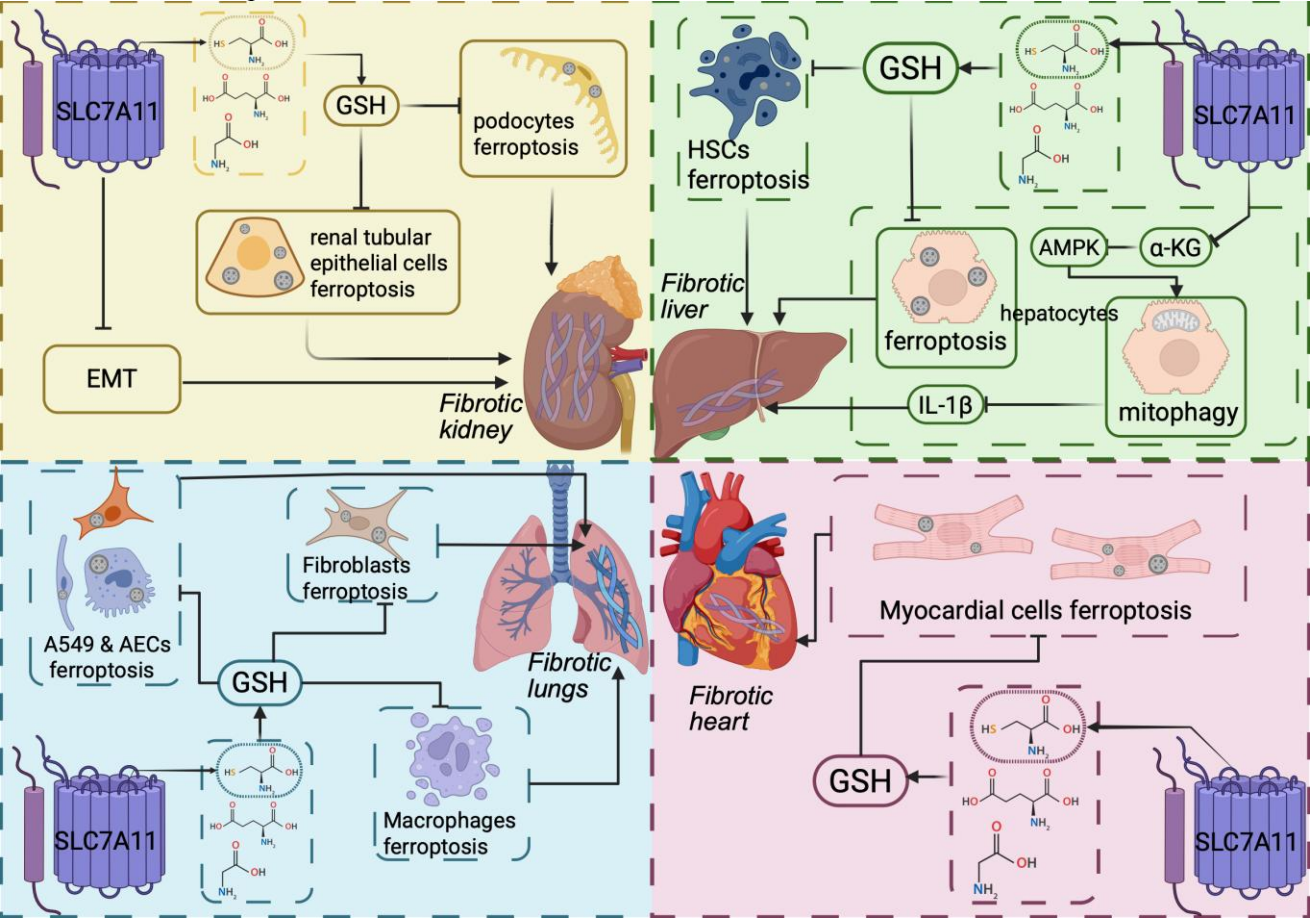
**The overall role of SLC7A11 in fibrosis of the kidneys, liver, lungs, and heart**. In the kidneys, SLC7A11 alleviates renal fibrosis by inhibiting ferroptosis in podocytes and renal tubular epithelial cells through GSH and may affect podocyte EMT. In the liver, SLC7A11 exacerbates liver fibrosis by inhibiting ferroptosis in HSCs and mitigates liver fibrosis by suppressing ferroptosis in hepatocytes and reducing IL-1β production and HSC activation. In the lungs, SLC7A11 inhibits ferroptosis in lung epithelial cells, fibroblasts, and macrophages, thereby preventing pulmonary fibrosis. In the heart, SLC7A11 reduces cardiac fibrosis primarily by inhibiting ferroptosis in cardiomyocytes.

Recent reports have highlighted the relationship between SLC7A11 expression and fibrosis in various other tissues. Yang et al. [194] discovered that SLC7A11 expression was downregulated in keloids, whereas Fer-1 application upregulated SLC7A11 in keloid fibroblasts, leading to reduced ECM accumulation and fibrosis. However, erastin had the opposite effect. SLC7A11 downregulation and ferroptosis have been observed in corneal scarring caused by bacterial keratitis. Furthermore, treatment with Fer-1 alleviates fibrosis [195]. Co-exposure to emamectin benzoate and microplastics increased the ROS levels in carp skeletal muscle, damaged mitochondrial function, and led to skeletal muscle atrophy and decreased muscle mass [196]. These findings suggest that SLC7A11 may be a key factor in modulating fibrotic processes in multiple organs and tissues. Figure 7 summarizes the role of SLC7A11 in fibrosis in the kidneys, liver, lungs, and heart. Table 2 provides an overview of drugs targeting SLC7A11 and their effects on fibrosis.

**Table 2:** Drugs that act on SLC7A11 and affect fibrosis.

Organ	Drug	Model	Target cells	SLC7A11-related pathways	Effects on SLC7A11	Effects on fibrosis	Ref
**Liver**	Aggregation-induced emission CN-based nanoparticles	CCl_4_	HSCs (targeted)	HIF-1α/SLC7A11	Downregulation	Mitigation	[102]
	Sorafenib*	CCl_4_	HSCs	HIF-1α/SLC7A11	Downregulation	Mitigation	[45]
	**Wogonoside**	CCl_4_	HSCs	SOCS1/SLCA11	Downregulation	Mitigation	[80]
		CCl_4_	HSCs	TRIM26 ubiquitination	Degradation	Mitigation	[50]
	**Ginsenoside Rh2**	CCl_4_	HSCs	IRF-1/SLC7A11	Downregulation	Mitigation	[81]
	**Chrysophanol**	HBx	HSCs		Downregulation	Mitigation	[82]
	**Acrylamide**		HSCs		Downregulation	Mitigation	[83]
	**Mesenchymal stem cell-derived exosomal miR-26a**		HSCs		Downregulation	Mitigation	[48]
	**Wild bitter melon**	LPS	HSCs		Downregulation	Mitigation	[85]
	**Ginsenoside Rb1**	CCl_4_	HSCs	BECN1/SLC7A11	Downregulation	Mitigation	[54]
	**Danshensu**		HSCs (LX-2 human and T6 rat HSCs)		Downregulation	Mitigation	[86]
	**Mori fructus aqueous extracts**	CCl_4_	Hepatocytes (HepG2)	Nrf2/ARE/SLC7A11	Upregulation	Mitigation	[88]
	**Luteolin**	CCl_4_	Hepatocytes (HepG3)		Upregulation	Mitigation	[91]
	Liraglutide*	T2DM	Hepatocytes		Upregulation	Mitigation	[89]
		NASH & SLC7A11 knockout mice	Hepatocytes	SLC7A11/mitophagy/IL-1β	Upregulation	Mitigation	[44]
	Antituberculosis drugs*		Hepatocytes	HIF-1α/SLC7A11	Downregulation	Aggravation	[46]
	**TBE-31**	NASH		Nrf2/ARE/SLC7A11	Upregulation	Mitigation	[74]
	**Artemisia Argyi essential oil**	BPA			Upregulation	Mitigation	[92]
	Glycyrrhetinic acid 3-O-mono-β-d-glucuronide (GAMG)	CCl_4_	Macrophages	IRF-1/SLC7A11	Downregulation	Mitigation	[96]
**Lungs**	Tuberostemonine	Bleomycin	HLFs		Upregulation	Mitigation	[120]
		Mo/Cd (sheep)			Downregulation	Aggravation	[125]
		Silicosis	Macrophages	Wnt5a/Ca^2+^	Downregulation	Aggravation	[124]
	**Astragalus and Panax notoginseng decoction**	Bleomycin		HIF-1α or EGFR (possibly)	Upregulation	Mitigation	[126]
**Kidney**	Formononetin	UUO- and folic acid-induced CKD	Primary mouse renal TECs	SMAD3/ATF3/ SLC7A11	Upregulation	Mitigation	[145]
	**As and PSNPs**		TECs (TCMK-1)		Downregulation	Aggravation	[156]
	**Vitexin**	DN	TECs (HK2)		Upregulation	Mitigation	[150]
	**Fer-1**	HN	TECs (HK2)		Upregulation	Mitigation	[152]
	**Fer-1**	CaOx	TECs		Upregulation	Mitigation	[147]
	**Kaempferitrin**	UUO	TECs		Upregulation	Mitigation	[148]
	**Hederagenin**	DN	TECs (HK2)	SMAD3/NOX4/ SLC7A11	Upregulation	Mitigation	[153]
	**Baicalein**	UUO	Podocytes (MPC-5)		Upregulation	Mitigation	[149]
	**Rhein**	DN	Podocytes (MPC-5)		Upregulation	Mitigation	[151]
		CaOx	TECs (HK2)	SOX4/EZH2/ SLC7A11	Downregulation	Aggravation	[35]
	Guanxining*	HF, transverse aortic constriction			Upregulation	Mitigation	[146]
	**Cu**	(Chicken)			Downregulation	Aggravation	[154]
**Heart**	Fer-1	Ang II	Cardiomyocytes (NRCMs)	Ang II/p53/SLC7A11	Upregulation	Mitigation	[47]
	**LuQi formula**	HF, transverse aortic constriction	Cardiomyocytes (NRCMs)	Nrf2/GPX4	Downregulation	Mitigation	[200]
	Resveratrol*	HF, transverse aortic constriction	Cardiomyocytes (hiPSC-CMs)	Sirt1/p53/SLC7A11	Upregulation	Mitigation	[171]
	**CF-exos-miR-23a-3p**	AF	Cardiomyocytes (H9C2)		Upregulation	Mitigation	[169]
	**MT**	LPS	Cardiomyocytes		Upregulation	Mitigation	[165]
	**Mitochondrial ALDH2**	AD			Upregulation	Mitigation	[170]
	**Beclin1 deficiency**	Alcohol-induced cardiac dysfunction			Upregulation (relative)	Mitigation	[164]
	Dexmedetomidine*	IRI			Upregulation	Mitigation	[172]
		IRI (after CPB)			Downregulation	Aggravation	[173]
	**Fuyu decoction**	HF (by ligating the left anterior descending coronary artery)		Nrf2/GPX4/SLC7A11	Upregulation	Mitigation	[174]
	Shenfu injection*	HF (isoproterenol induction)			Upregulation	Mitigation	[166]
	**tsRNA-5008a**	AF			Downregulation	Aggravation	[167]
	**TUDCA**	Obesity-induced cardiac dysfunction			Upregulation	Mitigation	[168]
**Others**	Fer-1	Keloid**	KFs		Upregulation	Mitigation	[194]
	**Fer-1**	*Pseudomonas aeruginosa* keratitis and CSSCs			Upregulation (relative)	Mitigation	[195]

“*” indicates that the drug has clinical applications beyond fibrosis treatment; “**” indicates that the referenced article involves clinical research, whereas the rest are preclinical studies. AD, Alzheimer’s disease; AF, atrial fibrillation; ALDH2, aldehyde dehydrogenase; As, arsenic; BPA, bisphenol A; CCl_4_, carbon tetrachloride; Cd, cadmium; CKD, chronic kidney disease; CN, carbon nitride; CPB, cardiopulmonary bypass; CSSCs, corneal stromal stem cells; DN, Diabetic nephropathy; EGFR, epidermal growth factor receptor; Fer-1, ferrostatin-1; HBx, hepatitis B virus X protein; HF, heart failure; HLF, human lung fibroblast; HN, hyperuricemic nephropathy; HSC, hepatic stellate cell; IRI, ischemia-reperfusion injury; KF, keloid fibroblast; LPS, lipopolysaccharide; Mo, molybdenum; MT, metallothionein; NASH, nonalcoholic steatohepatitis; NRCM, neonatal rat cardiac myocytes; PSNP, polystyrene nanoplastic; T2DM, type 2 diabetes mellitus; TBE-31, acetylenic tricyclic bis(cyano enone); TEC, tubular epithelial cell; TUDCA, tauroursodeoxycholic acid; UUO, unilateral ureteral obstruction

## Conclusion

Fibrosis is a pathological process involving multiple cell types, with each playing a distinct role in the onset and progression of fibrosis in different organs. Stellate cells, specifically HSCs and PSCs, influence the development of fibrosis in their respective organs. Under normal conditions, these cells function as lipid storage cells, but external stimuli such as oxidative stress or profibrotic factors such as TGF-β activate HSCs and PSCs, resulting in excessive ECM production and promoting the progression of liver and pancreatic fibrosis. Thus, the key to treating fibrotic diseases is to prevent HSCs from undergoing activation or to reduce ECM synthesis by these cells. In fibroblasts, metabolic pathways such as enhanced glycolysis, upregulated glutaminolysis, and increased fatty acid oxidation are notable profibrotic factors. Profibrotic factors, such as TGF-β, also exert an influence on fibroblast activation in organs, including the lungs, heart, and kidneys. However, no drugs aimed at these metabolic pathways have progressed to the clinical stage of testing. In addition to fibroblasts, various cells from different tissues and organs are engaged in the fibrosis of tissue and organs [11]. Inflammatory monocytes and resident tissue macrophages play crucial roles in tissue repair, regeneration, and fibrosis. Disruptions in macrophage function may lead to abnormal repair processes characterized by excessive inflammatory mediator and growth factor production, inadequate anti-inflammatory macrophage generation, or impaired communication between macrophages and other cell types. These disturbances can promote persistent injury and pathological fibrosis [197]. Parenchymal cells may also influence fibrosis initiation and progression directly or indirectly.

After understanding the key roles of various cells in the fibrosis process, SLC7A11, as a factor closely related to fibrosis, deserves in-depth exploration for its unique functions in different cells and organs. This review focuses on the effect of SLC7A11 on specific cell types in different organs and discusses its role in fibrotic diseases. At the cellular level, in HSCs, SLC7A11 can alleviate ferroptosis, supporting HSC activation and ECM production under the influence of profibrotic factors. SLC7A11 can protect hepatocytes from ferroptosis. In the parenchymal cells or fibroblasts of other organs, SLC7A11 provides protection against ferroptosis, thereby reducing ECM accumulation and alleviating organ fibrosis. SLC7A11 may also be associated with the inhibition of EMT initiation in epithelial cells. Furthermore, the expression of SLC7A11 on the surface of inflammatory cells may regulate immune cell function by modulating the inflammatory response. The influence of SLC7A11 on cell-to-cell interactions requires further investigation. SLC7A11 can reduce the production of inflammatory factors by regulating mitochondrial autophagy in hepatocytes, thereby preventing HSC activation and ECM production. SLC7A11 probably also influences immune cell function and modulates the activity of fibroblasts, stellate cells, and parenchymal cells via cytokine-dependent or -independent mechanisms.

SLC7A11 affects the progression of organ fibrosis by modulating cell function. In the liver, SLC7A11 controls the progression of liver fibrosis by reducing ECM production through the inhibition of hepatocyte ferroptosis and mitochondrial autophagy. It can also promote liver fibrosis progression by supporting HSC activation through the inhibition of HSC ferroptosis and may further affect liver fibrosis through inflammatory cells and inflammatory factors. All of these processes involve a complex regulatory network, which requires further exploration. In other organs, such as the lung, heart, and kidney, current studies suggest that SLC7A11 improves fibrosis by inhibiting parenchymal cell ferroptosis, thereby reducing ECM production. In some organs, it may also affect other cell types, such as inhibiting ferroptosis in macrophages and fibroblasts in the lung to improve fibrosis. In addition to ferroptosis, EMT may also be involved in fibrosis in the lung and kidney. SLC7A11 prevents fibrosis by inhibiting the initiation of EMT in epithelial cells.

At the organ level, the mechanisms by which SLC7A11 regulates fibrosis are complex and organ-specific. For example, upregulation or downregulation of SLC7A11 expression in liver fibrosis may yield different outcomes, and similar mechanisms may exist in other organs. This could serve as a direction for future research. Additionally, SLC7A11 may exert effects in multiple organs and could influence fibrosis through organ-to-organ interactions, such as those between the heart and kidney. The intricate interactions between organs regulated by SLC7A11 should also not be overlooked, especially when considering potential adverse effects in drug development. Future research should focus on the interactions of SLC7A11 between organs to further investigate its effect on the progression of fibrosis. SLC7A11 may also regulate interorgan and intersystem interactions through its effects on the immune system. Future research should explore how SLC7A11 modulates immune cells and their interactions with other cell types within organs, thereby improving our understanding of the role of the immune system in fibrosis. The involvement of the immune system in fibrosis progression is a critical avenue for future research, as SLC7A11 may influence fibrosis in multiple organs by altering immune cell behavior.

Non-ferroptotic mechanisms by which SLC7A11 influences fibrosis is an important avenue for future research. Current studies have identified that SLC7A11 can influence the progression of liver fibrosis through mitophagy. Other studies have only suggested that the downregulation of SLC7A11 is associated with both EMT and mitophagy, but they do not directly show a link between SLC7A11 and EMT. However, EMT and ferroptosis have been observed to occur simultaneously during fibrosis, suggesting they may share the same upstream regulatory factors. The core function of SLC7A11 is to regulate intracellular cysteine metabolism, thereby affecting the synthesis of GSH, which in turn affects cellular redox homeostasis. The ROS closely related to mitophagy are influenced by intracellular redox homeostasis and are therefore likely to be affected by SLC7A11. These potential mechanisms provide new directions for future research.

SLC7A11 exerts diverse effects on fibrosis in multiple organs through both ferroptosis-dependent and non-ferroptosis-dependent mechanisms. Its multifaceted involvement suggests that targeting SLC7A11 may offer a promising strategy for future treatment of fibrosis. Further investigation into how SLC7A11 affects fibrosis at the cellular, organ, and immune system levels is crucial for developing targeted therapies, particularly for liver fibrosis, and advancing our understanding of its potential as a therapeutic target in fibrotic diseases.
